# Consumer behaviour survey for assessing exposure from consumer products: a feasibility study

**DOI:** 10.1038/s41370-018-0040-2

**Published:** 2018-05-23

**Authors:** Klaus Schneider, Selina Recke, Eva Kaiser, Sebastian Götte, Henrike Berkefeld, Juliane Lässig, Thomas Rüdiger, Oliver Lindtner, Jan Oltmanns

**Affiliations:** 1 FoBiG, Forschungs- und Beratungsinstitut Gefahrstoffe GmbH, Klarastr. 63, 79106 Freiburg i. Br., Germany; 2 aproxima, Gesellschaft für Markt- und Sozialforschung Weimar mbH, Schillerstraße 10, 99423 Weimar, Germany; 3 BfR, Bundesinstitut für Risikobewertung, Diedersdorfer Weg 1, 12277 Berlin, Germany

**Keywords:** REACH, Consumer survey, Frequency, Duration, Amount, Exposure scenarios

## Abstract

Evaluating chemical exposures from consumer products is an essential part of chemical safety assessments under REACH and may also be important to demonstrate compliance with consumer product legislation. Modelling of consumer exposure needs input information on the substance (e.g. vapour pressure), the product(s) containing the substance (e.g. concentration) and on consumer behaviour (e.g. use frequency and amount of product used). This feasibility study in Germany investigated methods for conducting a consumer survey in order to identify and retrieve information on frequency, duration, use amounts and use conditions for six example product types (four mixtures, two articles): hand dishwashing liquid, cockpit spray, fillers, paints and lacquers, shoes made of rubber or plastic, and ball-pens/pencils. Retrospective questionnaire methods (Consumer Product Questionnaire (CPQ), and Recall-Foresight Questionnaire (RFQ)) as well as protocol methods (written reporting by participants and video documentation) were used. A combination of retrospective questionnaire and written protocol methods was identified to provide valid information in a resource-efficient way. Relevant information, which can readily be used in exposure modelling, was obtained for all parameters and product types investigated. Based on the observations in this feasibility study, recommendations are given for designing a large consumer survey.

## Introduction

Evaluating chemical exposures from consumer products is an essential part of chemical safety assessments under the European chemicals legislation REACH (Regulation (EC) No 1907/2006) and may also be important to demonstrate compliance with consumer product legislation. Consumer products may be articles or mixtures according to the definition given in the above mentioned REACH regulation. Only few specific groups of consumer products have their own legislation to ascertain absence of effects on human health (e.g. Directive 2009/48/EC (Toy Safety Directive), Regulation (EC) No 1223/2009 (EU Cosmetic Regulation). In contrast, the REACH Regulation requires manufacturers and importers of hazardous substances eventually included in consumer products to assess exposure and risk from using these products in a chemical safety report, if a substance is manufactured or imported in quantities >10 tons/year. As thousands of substances can be concerned, REACH is a powerful instrument to improve consumer safety and public health.

Direct exposure to chemicals from consumer products is rarely measured. Biomonitoring can give helpful information but in many cases is unable to link a product to the biomarker measured. Therefore, exposure modelling is often the method of choice to assess consumer exposure. Frequently applied software tools for assessments under REACH are ConsExpo, which was developed by the Dutch National Institute for Public Health and the Environment (RIVM) and recently established as a web-based tool (http://www.rivm.nl/en/Topics/C/ConsExpo), and the consumer exposure module of ECETOC TRA, as included in the European Chemicals Agency’s CHESAR tool (https://chesar.echa.europa.eu/).

Typically, a consumer exposure assessment needs input information on the substance (e.g. vapour pressure), the product(s) containing the substance (e.g. concentration) and on consumer behaviour (e.g. use frequency and amount of product used). The latter category of information provides special challenges for a registrant of a chemical under REACH, as it is linked to the private end user at the very end of the supply chain. Key parameters in that respect are frequency and duration of product use, amounts used, location and conditions of use. So-called fact sheets developed for ConsExpo summarise the data available, which can be used to inform the tool and the implemented algorithms with regard to these key parameters. Fact sheets are available for some groups of products in the area of cleaning products, toys, do-it-yourself products and paints [1–4]. Tools use different default values (i.e. pre-set values for parameters, proposed to be used in different scenarios in absence of better information), based on assumptions, expert judgements or data [5].

Some efforts have been made so far to improve the database on consumer behaviour. For example, in the EPHECT (“Emissions, Exposure Patterns and Health Effects of Consumer Products in the EU”) project [6–8] information on 16 product types from 10 European countries was surveyed. These product types include several cleaning products, cosmetics, biocides and air fresheners (none of these product types are addressed in this study). Garcia-Hidalgo et al. [9] investigated the use pattern of cleaning and personal care products in Switzerland. This study used a comprehensive questionnaire to obtain data on frequency and duration (for household cleaning products) and further parameters including amounts used for personal care products. Also, industry sectors engage in surveys for improving the database for their supply chain, but—by their very nature—cover only specific groups of products [10–13]. Furthermore, previous surveys partly failed to identify relevant consumer information in a way suitable for chemical exposure assessment. For example, exposure frequency was expressed as the percentage of individuals using specific products with a certain frequency in the EPHECT project [6], whereas average values, medians and higher percentiles are required for exposure assessments using tools such as ConsExpo or ECETOC TRA. Also Garcia-Hidalgo et al. [9] reported detailed data, differentiated according to age groups, but presented results as percentage of responders falling into a specific category (e.g. percentage of users using dishwashing detergents from 31 to 40 min/day), which makes it difficult to use the data for exposure modelling.

Moreover, a rough survey of online inventories of a chemist’s shop and a building supply store indicates that there are hundreds of different product types consisting of mixtures on the market. The number of relevant consumer products increases substantially, if also articles giving rise to chemical exposure are considered. Existing software tools and the implemented exposure models address only a small part of the product types in sufficient detail.

Considering the extent of the existing data gaps, this feasibility study was initiated. Although it specifically aims at producing data, which can directly be used for (deterministic) consumer exposure assessments under the EU legislation REACH (Regulation (EC) No 1907/2006), such information may also provide helpful input to aggregate (i.e. considering multiple sources) deterministic or probabilistic exposure assessments using tools such as PACEM [14, 15] or CRÈME RIFM [16].

The feasibility study has a two-fold objective:

1. identify relevant consumer behaviour information for six example consumer product types (four mixtures and two types of articles) and check the applicability for exposure evaluations,
2. apply, critically assess and compare the usefulness of various survey methods and recommend methods for conducting a large consumer behaviour survey.

This publication reports the results of this feasibility study with a focus on the first objective. A parallel publication (Recke et al. 2018, in preparation) provides a detailed analysis of methodological observations and a recommendation of methods for a large-scale survey.

## Methods

### Product types and enquired information

“Product type” as used in this paper refers to a group of products, which concur in their use conditions and type of application (example: “hand dishwashing liquid” is a product type, to which products of various brands belong).

Product types for inclusion in the feasibility study were selected according to the following criteria: they must represent mixtures and articles, must include often and rarely used product types, should include different types of applications and exposure routes (dipping, spraying, use of tools for application), and should include at least one well-known product type to allow for comparison with literature data.

The survey aimed at retrieving data for the following parameters: frequency and duration of product use, amounts used (for mixtures) or weight (for articles) as well as location and conditions of use. Based on these criteria, the following product types were selected: hand dishwashing liquid, cockpit spray (for cleaning the car interior), filler (powder to be suspended in water for filling small holes or crevices in walls, floors, etc.); paints and lacquers (except wall paints; actually consist of different product types and exposure conditions, from painting tiny models to arbours); shoes made of rubber or plastic (such as beach sandals; except Wellington boots); and ball-pens and pencils.

For all product types, the input information required in the exposure estimation tools ConsExpo and ECETOC TRA was identified. In order to compare survey methods (e.g. Consumer Product Questionnaire (CPQ), and Recall-Foresight Questionnaire (RFQ), see below), information needs were assigned to more than one survey method (if applicable). Information requests were aimed to be designed in a way that makes responses directly usable in exposure estimation tools.

### Survey methods

For reasons of cost-effectiveness, only methods not requiring home visits were considered. A panel design (panel: a pool of people who have agreed to participate in surveys) was used, which allows repeated interviews of the same people either with the same or with a different survey method. After weighing the pros and cons of all possible methods, a longitudinal combined telephone/online panel was chosen and established. The aim was to compare four different field methods—12-month retrospective questionnaire, 4 weeks retrospective questionnaire, protocol and video camera—in terms of 10 criteria related to the usability for assessing exposure, validity and completeness of the answers, ease of use and costs.

The survey consisted of two levels—level 1 included questionnaires (CFQ and RFQ, see below) for all participants and level 2 containing so-called “tasks” (documentation of information in protocols and video recordings) for participants planning to use any of the product types in question. Participants were recruited in Germany from panel pools owned by aproxima Gesellschaft für Markt- und Sozialforschung Weimar mbH (Weimar, Germany) and were free to decide whether to run in the telephone or online track. At first, they had to fill in a “Consumer Product Questionnaire (CPQ)” with questions regarding whether they used certain product types, the use frequency over the last 12 months and other use conditions. A few weeks later, the CPQ participants were invited to answer to a second questionnaire, called “Recall-Foresight Questionnaire (RFQ)”. Both questionnaires are available as supplemental material (SI). The aim of the RFQ was to gather information on usage behaviour over the past 4 weeks. For a large part, the RFQ contained the same questions as the CPQ to allow a comparison between the two methods. Furthermore, the RFQ was used to recruit participants for the tasks (level 2). Any respondent intending to use one or more of the given product types in the near future was asked to either fill out a protocol (which consisted of a printed list of questions to be answered in writing, available as SI) during the next usage or to do a video of the usage. In case they agreed, they received a protocol and—in the video group—a small action camera. Each protocol or video should cover one event only.

The aim of the feasibility study was not to create representative data but method testing. Neither were the participants recruited to match any population criteria nor was any weight applied to achieve representativeness.

The field phase of the study lasted from August 2016 to February 2017. For more details on the methods, see Recke et al. (2018, in preparation).

The number of non-responders and/or non-users varied from question to question within one product type. Therefore, slight deviations in the number of users for one product type in the same questionnaire might occur.

### Calculation and statistics

#### Distributions for categorical responses

In case answers were designed as categorical responses (e.g. frequency questions: <1 per month, 1–3 times per month, etc.), numerical values were calculated by using arithmetic means for ranges (e.g. 1–3 times per month was calculated as 2 times per month); <1 per month was calculated as 0.5 times per month.

#### Statistical analyses

Statistical values for distributions were calculated using Analyse-it for Microsoft Excel 4.65.3 (Analyse-it Software, Ltd., Leeds, UK, 2017). This software was also used for testing the dependency between parameters. Spearman’s rank-order correlation was used, since it requires monotonic but not normally distributed values. The level of significance was set to *p* < 0.05.

## Results

### Representativeness of data

The CPQ’s total sample consisted of 4139 persons, 1186 of them were contacted via telephone and 2953 via e-mail. Overall, 631 of the telephone sample and 593 of the online sample took a valid interview. The gender distribution of participants is close to the actual distribution in the German population (see Table 1). It is obvious that more women participated in the telephone survey, while relatively more men participated online. Concerning the age distribution, the younger age groups, which are difficult to reach via telephone surveys, are more frequently represented in the online sample, whereas the over 70s are still underrepresented. Although this feasibility study did not aim to achieve this goal, with the combination of telephone survey and online survey the population of participants comes close to being representative.

**Table 1:** Representativeness of participants from the Consumer Product Questionnaire (CPQ)

Age/gender	German-speaking population of Federal Republic of Germany (18 and older) (%)	Participants of the study (%)	Participants of the telephone survey (%)	Participants of the online survey (%)
18–30 years	18.5	13.0	7.2	19.3
31–40 years	14.6	15.3	11.6	19.3
41–50 years	17.4	18.0	18.2	17.9
51–60 Years	18.4	23.1	26.8	19.1
61–70 years	13.2	18.5	20.2	16.6
71 years and older	17.9	12.1	16.1	7.8
Men	48.9	49.8	47.1	52.9
Women	51.1	50.2	52.9	47.1

### Suitability of methods

Questionnaire methods (CPQ and RFQ) proved applicable and yielded similar results for categorical parameters such as use conditions (use of gloves, type of material, etc.). Also, information on frequency could be obtained similarly by both methods, although the enquiry period of 4 weeks in the RFQ was too short to obtain meaningful results in the case of infrequently used product types (cockpit spray, fillers). For articles, weighing tasks could be integrated in the CPQ with suitable response rates (24% and 48% for shoes and pens/pencils, respectively). Therefore, in the case of articles CPQ was the method of choice to obtain information on all parameters requested.

For mixtures, parameters which require measurements (use amount, duration of event) were investigated with protocols and video documentation. The protocol method proved to be advantageous, as willingness to participate was much higher than for video cameras and the percentage of valid protocols among those returned was high. For cameras, in addition to the recruitment difficulties, technical problems (camera not started, inappropriate camera angle, quantitative scales not recognisable) led to a low number of valid documentations. In conclusion, video documentation is considered a suitable method only for special applications, where visual information is mandatory to understand the use conditions (for example, for a product which can be applied in many different ways). Owing to the limited number of video protocols obtained, results are not reported in section “Exposure parameters”.

### Exposure parameters

**Fig. 1:**
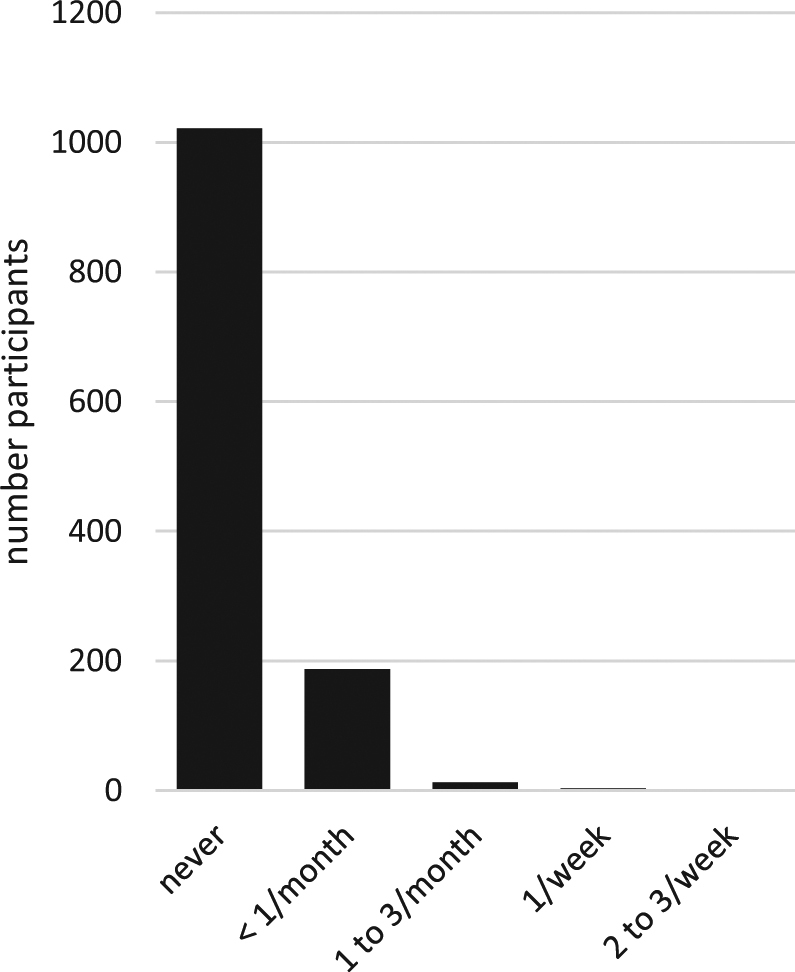
Frequency of cockpit spray use obtained from CPQ—all responders. (single column fitting image)

For all product types and for most of the parameters, the feasibility study yielded important quantitative information. As an example, Fig. 1 graphically presents data on the use frequency of cockpit spray as obtained from the CPQ survey. Statistical values were derived from the empirical distributions obtained as explained in “Methods”.

The following sections present and discuss the results per product type as obtained from the CPQ and the protocols. Except information on frequency for infrequently used product types (see section “Suitability of methods”), values obtained from RFQ are close to those from CPQ and are not reported here.

#### Hand dishwashing liquid

Table 2 provides statistical key values for the distributions obtained for use frequency, duration of use and amounts used per event.

**Table 2:** Hand dishwashing liquid: information on use frequency, duration, and amounts

Parameter	Respondents	Unit	*N*	Min.	5th perc.	25th perc.	AM ± SD	Median	75th perc.	90th perc.	95th perc.	Max.
Use frequency^a^	All	1/day	1212	0.0	0.0	0.4	0.8 ± 0.8	0.6	0.9	1.9	2.8	11.1
	Frequent users^c^	1/day	1084	0.1	0.1	0.4	0.9 ± 0.8	0.7	0.9	1.9	2.8	11.1
Duration per event^b^		Minutes	64	1.0	2.0	6.0	17.3 ± 34.0	10.0	17.0	30.0	46.2	345.0
Amount used per event^b^		Grams	56	<0.05	1.0	2.0	5.5 ± 5.2	4.0	7.0	12.0	14.0	42.0

*AM* arithmetic mean, *SD* standard deviation

^a^From CPQ

^b^From protocols

^c^More than 15× per year

Use frequency was determined in a different way compared to other product types. In the CPQ, panel members were asked for the number of days per week they are using hand dishwashing liquid and for the number of dishwashing events per day. For all individual data sets, both responses were multiplied to obtain the use frequency per individual. In order to comply with the definition of frequent use in ECHA’s Guidance on Consumer Exposure Assessment [17] for calculating long-term use frequency, only those users applying the product more often than 15 times per year were considered.

In addition, the CPQ surveys asked participants whether they use hand dishwashing liquids for other purposes than dishwashing. In all, 48% gave a positive response in the CPQ. The main other uses were (in decreasing frequency) surface cleaning, hand washing, cleaning of eyeglasses, cleaning of windows and removal of stains.

Furthermore, participants were asked to give information on the room, in which the product is used (99%: kitchen). In the protocols, participants were asked to estimate the size of the room of application. The 22 protocols containing this information identified a range of room sizes from 3 m^2^ to 36.4 m^2^ (median: 9 m^2^, 75th perc.: 12 m^2^; 90th perc.: 29.3 m^2^).

#### Cockpit spray

Out of the 1224 participants in the CPQ, 203 individuals (17%) used cockpit spray in the past 12 months prior to the survey for cleaning the automobile interior and 63% used spray cans (compared to 36% using products in trigger sprays). Table 3 gives the information on frequency, duration of application and amount used per event, as obtained from the CPQ and the protocols.

**Table 3:** Cockpit spray: information on use frequency, duration, and amounts

Parameter	Unit	*N*	Min.	5th perc.	25th perc.	AM ± SD	Median	75th perc.	90th perc.	95th perc.	Max.
Use frequency^a^											
All	1/month	1224	0.0	0.0	0.0	0.1 ± 0.5	0.0	0.0	0.5	0.5	10.7
Users	1/month	203	0.5	0.5	0.5	0.7 ± 0.9	0.5	0.5	0.5	2.0	10.7
Duration per event^b^	Minutes	13	14.0	14.0	18.7	44.9 ± 37.6	25.0	65.0	106.7	140.0	140.0
Amount used per event^b^	Grams	13	10.0	10.0	22.0	48.5 ± 56.7	30.0	51.0	131.0	225.0	225.0

*AM* arithmetic mean, *SD* standard deviation

^a^From CPQ

^b^From protocols

Moreover, information on the location of use was obtained: 85% of the users used the products outdoors, 8% in the garage, and 5% in carports. In all, 96% of users stated that they applied the product with car doors opened, and half out of the remaining 4% confirmed to have windows open during application. From this, we conclude that a high percentage of users apply the cockpit spray under conditions of good ventilation.

#### Fillers

In all, 32% (386 out of the 1224) of the participants in the CPQ stated to have used fillers at least once in the past 12 months prior to the survey. Again, Table 4 provides information on frequency, duration and amounts used. For this product, participants were asked in the protocols to measure the application time as well as the time required for mixing the solid product with water to make it ready for use.

**Table 4:** Fillers: information on use frequency and duration

Parameter	Unit	*N*	Min.	5th perc.	25th perc.	AM ± SD	Median	75th perc.	90th perc.	95th perc.	Max.
Use frequency^a^											
All	1/month	1224	0.0	0.0	0.0	0.3 ± 0.8	0.0	0.5	0.5	0.5	19.3
Users	1/month	386	0.5	0.5	0.5	0.8 ± 1.2	0.5	0.5	0.5	2.0	19.3
Duration of mixing phase^b^	Minutes	10	1.0	1.0	2.0	3.6 ± 2.6	2.5	5.0	8.2	10.0	10.0
Use duration per event^b^	Minutes	10	5.0	5.0	9.7	32.4 ± 22.6	33.0	45.8	67.7	75.0	75.0

*AM* arithmetic mean, *SD* standard deviation

^a^From CPQ

^b^From protocols

Table 4 does not contain information on amounts used, as only six protocols were returned with valid information for this parameter and no distribution could be calculated from such a small number. In these six protocols, a wide range (30–4183 g) of product amount used was reported.

According to the results from the questionnaire, fillers are used mainly (90%) indoors, but no specific room dominates. The vast majority of respondents (97%) uses tools (in decreasing order of frequency): spatula only, spatula plus ladle, and ladle only.

#### Paints and lacquers

Participants were asked to record any type of use of paints or lacquers. In the questionnaire, 58% (716/1224 participants) reported to have not used paints or lacquers in the 12 months before the survey. In all, 20% of users painted a picture; 78% painted an object, among them large objects such as arbours, fences and balconies, but also small objects like models or other small pieces; and the remainder painted objects not belonging to certain categories or did not provide information on the type of object. Owing to the vast range of object sizes, duration and use amounts vary greatly (see Table 5).

**Table 5:** Paints and lacquers: information on use frequency, duration, and amounts

Parameter	Unit	*N*	Min.	5th perc.	25th perc.	AM ± SD	Median	75th perc.	90th perc.	95th perc.	Max.
Use frequency^a^											
All	1/month	1216	0.0	0.0	0.0	0.5 ± 1.7	0.0	0.5	2.0	2.0	19.3
Users	1/month	498	0.5	0.5	0.5	1.4 ± 2.4	0.5	0.5	2.0	4.3	19.3
Painting a picture	1/month	101	0.5	0.5	0.5	1.4 ± 2.1	0.5	4.3	4.3	9.0	19.3
Furniture	1/month	104	0.5	0.5	0.5	0.8 ± 0.7	0.5	0.5	4.3	4.3	4.3
Model making and other small parts	1/month	59	0.5	0.5	0.5	1.9 ± 3.6	0.5	2.0	4.3	9.0	19.3
Doors, windows and stairs	1/month	41	0.5	0.5	0.5	0.8 ± 0.8	0.5	0.5	4.3	4.3	4.3
Cabins and bowers	1/month	18	0.5	0.5	0.5	0.7 ± 0.5	0.5	0.5	1.6	2.0	2.0
Items made of wood-like picture frames	1/month	71	0.5	0.5	0.5	1.3 ± 2.2	0.5	0.5	2.1	4.9	10.9
Fences and balconies	1/month	25	0.5	0.5	0.5	0.9 ± 0.7	0.5	2.0	2.0	2.0	2.0
Car and car components	1/month	17	0.5	0.5	0.5	1.2 ± 2.5	0.5	0.5	2.0	9.0	10.9
Duration per event^b^	Minutes	23	2.0	11.0	34.0	94.3 ± 103.9	55.0	139.2	180.0	337.5	495.0
Amount used per event^b^	Grams	23	1.7	3.9	34.2	237.8 ± 274.6	82.0	388.8	620.0	850.0	1000.0

*AM* arithmetic mean, *SD* standard deviation

^a^From CPQ

^b^From protocols

When asked for the location of application, the living room predominates, but, of course, depending on the object painted, various rooms as well as outdoor locations were mentioned.

In protocols, participants were asked for information on keeping doors or windows closed during application. Out of the 20 participants using paints or lacquers indoors, 18 (90%) stated to have either doors or windows or both open during application.

#### Ball-pens or pencils

This group of articles consists of any hand-held writing object, such as pencils or ball-pens. A special feature of this group of articles is the potential for oral exposure due to mouthing.

In the case of articles, only CPQs and RFQs were used. In order to avoid more laborious protocols, the task to weigh the articles with available kitchen scales was included in the questionnaires. Table 6 summarises the information obtained for frequency and duration of use as well as the weight of the objects. Only one individual did not use a writing object over the past 12 months. Out of the 1224 participants, 1216 provided further information.

**Table 6:** Ball-pens/pencils: information from the CPQ on use frequency, duration, and weight

Parameter	Unit	*N*	Min.	5th perc.	25th perc.	AM ± SD	Median	75th perc.	90th perc.	95th perc.	Max.
Use frequency											
All	1/day	1224	0.0	0.4	0.9	0.9 ± 0.2	0.9	0.9	0.9	0.9	0.9
Users	1/day	1223	0.02	0.4	0.9	0.9 ± 0.2	0.9	0.9	0.9	0.9	0.9
Duration per event	Minutes	1216	1.0	3.0	10.0	61.0 ± 83.0	30.0	60.0	140.3	240.0	640.0
Weight of writing utensils	Grams	581	2.0	4.0	9.0	13.7 ± 11.1	11.0	15.0	22.0	30.0	150.0

*AM* arithmetic mean, *SD* standard deviation

Approximately 48% (581/1216 participants) agreed to weigh the writing object in use and provided valid weight data with the CPQ. Additionally, participants provided information on the material of the writing objects (80% plastic, 11% metal, 7% wood) and on the location of use (at the workplace: 46%, in the home office: 20%, in the living room: 20%, in the kitchen: 8%). Obviously, in the case of writing objects it is difficult to differentiate between private use and use at the workplace.

About 15% of participants (187/1216) confirmed to touch their writing object with the mouth during use. The self-reported range of durations for touching the pens with the mouth was from 0.1 to 150 min/day (median: 3 min/day). As mouthing behaviour is an unconscious action, self-reported values need to be considered with care.

#### Shoes made of plastic or rubber

As with paints and lacquers, the article group of “shoes made of plastic or rubber” consists of many different product types. The kinds of shoes made of these materials vary from flip-flops to more closed slippers or clogs. Wellington boots and sports shoes were excluded. Owing to different types of shoes, responses to the questions vary over a wide range. In all, 58% (708/1224) of the participants confirmed to use shoes made of plastic or rubber. Again, participants were asked to weigh their shoes. In this case, only 187 out of the 708 (26%) agreed to weigh their shoes. The results for the relevant parameters are reported in Table 7.

**Table 7:** Shoes made from rubber or plastic: information from the CPQ on use frequency, duration, and weight

Parameter	Unit	*N*	Min.	5th perc.	25th perc.	AM ± SD	Median	75th perc.	90th perc.	95th perc.	Max.
Use frequency											
All	1/day	1223	0.0	0.0	0.0	0.2 ± 0.3	0.02	0.4	0.6	0.9	0.9
Users	1/day	704	0.02	0.0	0.1	0.3 ± 0.3	0.1	0.4	0.9	0.9	0.9
Duration per event	Hours	704	0.5	0.5	0.5	2.8 ± 2.7	1.5	3.0	7.0	9.0	13.0
Weight of shoes made from rubber or plastic	Grams	172	10.0	51.9	99.4	152.4 ± 92.3	133.0	190.0	251.7	302.5	700.0

*AM* arithmetic mean, *SD* standard deviation

Furthermore, information on the type and on the material of the shoes (rubber 45%, plastic 35%, mixed materials 3%) was obtained. Also, participants were asked to provide information on whether shoes have lining, are worn with socks and/or are worn more frequently during specific seasons.

As expected, use behaviour varied depending on the type of shoe. Therefore, a clear definition of types of shoes and a stratified survey is mandatory for obtaining suitable results for individual shoe types.

#### Other use conditions

The CPQ and the protocols asked for additional product-type-specific information: use of gloves during product application, reading and following safety instructions, and whether users always use the same brand. Table 8 gives an overview on this information for all product types consisting of mixtures. The percentage of consumers using gloves, reading and following instructions, or using the same brand varies between products. However, for none of the product types the percentages of glove users and of users following instructions are high enough to deviate from the default procedure and to use the less conservative assumption that risk mitigation measures apply.

**Table 8:** Information on other use conditions (in percentage of participants) for product types consisting of mixtures (from CPQs, if not indicated otherwise)

Use condition	Dishwashing liquid		Cockpit spray		Filler		Paints and lacquers	
	Yes^a^	No	Yes	No	Yes	No	Yes	No
Wear gloves during use	3.1	91.6	5.9	85.2	17.1	70.1	41.7^b^	58.3^b^
Read instructions	17.4^b^	81.0^b^	52.2	46.3	70.8	29.2	75.0^b^	25.0^b^
Follow instructions	31.3^b^	65.1^b^	76.4	21.2	82.1	17.1	95.8^b^	4.2^b^
Use always same brand	43.2	56.2	30.5	63.5	29.2	65.7	33.3^b^	66.7^b^

^a^Sum of yes and no answers do not sum up to 100% due to non-responders or “don’t know” answers

^b^Data from protocols without camera

#### Parameter dependency

**Fig. 2:**
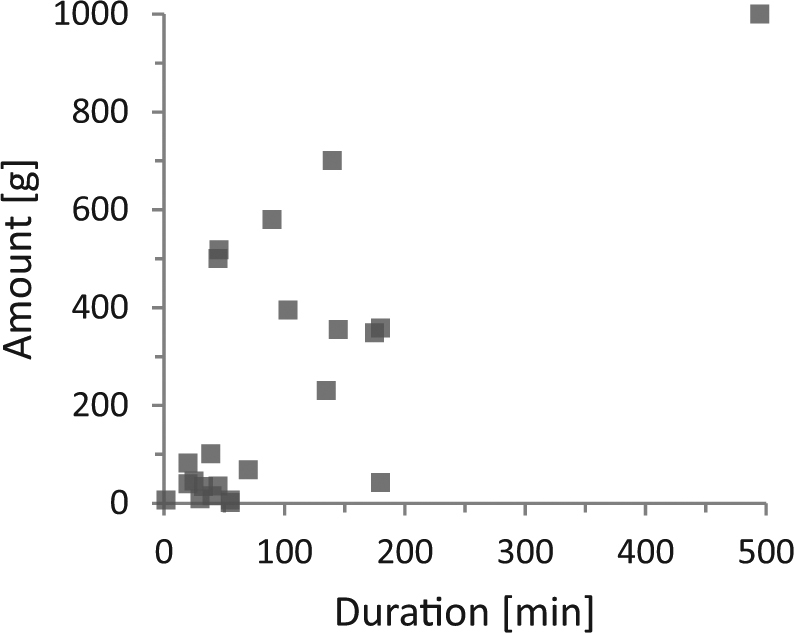
Correlation of amount used and use duration for paints and lacquer. (single column fitting image)

Exposure assessment aims at a conservative but realistic estimate of exposure, often defined as a specific, higher percentile of the users. Combining high percentile values of individual parameters by multiplication would lead to very high percentiles of users included. This is especially true where parameters are not independent. Therefore, possible dependencies between parameters were analysed for possible correlations, when the values were retrieved by the same method. Table 9 provides the results of the correlation test of the individual data pairs for the parameters use duration and amount for the four mixtures obtained with the protocol method. Figure 2 is a graphical presentation of the data set for hand dishwashing liquid.

**Table 9:** Statistical analysis of dependency of parameters amount and duration obtained from protocols

Correlation	*N*	Spearman’s *r*s	*p*-Value
Hand dishwashing liquid	168	0.48	<0.0001^a^
Cockpit spray	13	0.26	0.46
Fillers	6	0.09	0.87
Paints and lacquers	23	0.56	0.006^a^

^a^Statistically significant (*p* < 0.05)

Statistically significant correlations between duration and amounts used were found for dishwashing liquid as well as paints and lacquers, whereas no correlation could be detected for cockpit spray and fillers. The number of protocols obtained for the latter two product types was low. No clear correlation could be found for individual data on use frequency and duration obtained from the CPQ (data not shown).

## Discussion

### Observations from feasibility study

Only few reliable data sources exist for consumer behaviour data for the many product types in use. Recently, in the EPHECT project, consumer behaviour data for participants from 10 EU countries on 16 different product types were published [6–8]. However, some of the data retrieved by EPHECT were difficult to interpret. For example, exposure duration is reported in the EPHECT project as the percentage of participants falling into a specific category (e.g. once per month). Amounts for cleaning products are reported as the number of caps per use event, without indicating cap size or density of products. Similarly, Garcia-Hidalgo et al. [9] reported use frequency and duration for dishwashing detergents as percentage of responders falling into certain categories. These data are difficult to compare with the outcome of this study, which reported frequency and use amounts as distributions of numerical values with units “times per month” and “grams of product”, respectively. This has been done as this study aimed at retrieving information ready to use for exposure assessment in widely used consumer exposure models such as ECETOC TRA (also as part of ECHA’s tool for creating chemical safety reports, CHESAR) or ConsExpo. But in their discussion part, Garcia-Hidalgo et al. [9] mention that “average” use frequency for dishwashing detergent in their study was 5.4 times per week (0.77 per day) and “average” duration was 19 min. These values are close to the arithmetic mean values observed in our study (Table 10).

**Table 10:** Comparison of exposure values obtained for dishwashing liquid with data from literature

Parameter	Source	Study population from
Frequency [AM **±** SD (events per day)]		
0.9 ± 0.8	This study (*n* = 1084 users)	Germany
0.63 ± 0.79	[22] (*n* = 45 users with 596 events)	Netherlands
0.93 ± 0.64	[23] (*n* = 115 users)	France
2 (“typical value”)	[11] (no information on number of participants available)	Presumably EU
2.35	[24] (value extracted from figure, *n* = 2741 participants (82% used the product))	South Korea
0.77 (“average”)	[9] (*n* = 759)	Switzerland
Duration [AM ± SD (min per event)]		
18.6 ± 34.0	This study (*n* = 64 participants with 192 events)	Germany
11.0 ± 5.0	[22] (*n* = 45 participants with 596 events)	Netherlands
9.27	[24] (*n* = 2741 participants)	South Korea
30.0 (“typical value”)	[11] (no information on the number of participants available)	Presumably EU
7.0 ± 5.0	[25] (*n* = 28 participants)	Lebanon
19 (“average”)	[9] (*n* = 759)	Switzerland
Amount [AM ± SD (g)]		
5.5 ± 5.2	This study (*n* = 56 participants with 168 events)	Germany
7.0 ± 6.0	[19] (*n* = 27 participants)	Netherlands
5.8	[24] (*n* = 2741 participants)	South Korea
2.0 ± 1.2 (mL)	[25] (*n* = 28 participants)	Lebanon

*AM* arithmetic mean, *SD* standard deviation

For two of the product types investigated, hand dishwashing liquid and paints and lacquers, further literature data exist that can be compared with the results obtained.

Values on frequency and use amounts obtained for hand dishwashing liquid in this study are comparable to results from previous European studies, whereas a somewhat higher figure for duration of dishwashing events was obtained here. The industry sponsored HERA survey produced higher values (defined as “typical values” without further information) for frequency and duration than any other European survey. For use amounts, only a range of 3–10 g per task is given in the HERA survey [11]. A study from South Korea resulted in higher figures for frequency but a lower duration (Table 10). Whether socio-cultural differences between study populations from different countries or continents are a source of differences is unknown.

Data are difficult to compare in the case of paints and lacquers, as many different exposure situations are comprised in the data set of this feasibility study, from painting tiny models to large objects. Westat Inc. [18] report lower frequencies, longer duration and higher amounts used per event, but these data most certainly reflect a different use situation with painting large wall surfaces. The study by Weegels [19], based on 10 participants concluded on a higher use frequency but lower durations and amounts used, compared to average values in this study (see Table 11).

**Table 11:** Comparison of exposure values obtained for paints and lacquers with data from literature

Parameter	**Source**	Study population from
Frequency [AM SD (events per month]		
1.3 ± 2.4	This study (*n* = 498 users)	Germany
0.33 ± 1.73	[18, 26] (*n* = 1794 users of latex paint	United States
0.47 ± 1.92	[18, 26] (*n* = 735 users of oil paint)	United States
4.2 ± 13.5	[19] (*n* = 10 users with 30 events)	Netherlands
Duration [AM ± SD (min per event)]		
94.3 ± 103.9	This study (*n* = 23 participants)	Germany
295.08 ± 476.11	[18, 26] (*n* = 1769 participants, latex paint)	United States
194.12 ± 345.68	[18, 26] (*n* = 726 participants, oil paint)	United States
25.00 ± 19.00	[19] (*n* = 9 participants, alkyd/acryl paints)	Netherlands
Amount [AM ± SD (g)]		
237.8 ± 274.6	This study (*n* = 23 users)	Germany
5471.46 ± 8799.69	[18, 26] (*n* = 1759 participants, latex paint)	United States
3052.96 ± 8599.83	[18, 26] (*n* = 698 participants, oil paint)	United States
44.00 ± 26.00	[19] (*n* = 10 participants)	Netherlands

*AM* arithmetic mean, *SD* standard deviation

For fillers, only one study was found in the literature reporting data on use frequency and duration. This study [20] is based on data for four individuals only. Two of them used putty to smooth a wooden surface; two others filled holes in a stone wall. These four activities lasted approximately 3–9 min per event and 5–31 g (filling of small holes in wooden board) were consumed. Longer use periods with more product used are reported in our study (arithmetic mean of use duration: 32.4 min, see Table 4). A higher number of responses is necessary to establish meaningful distributions.

Table 12 provides a comparison of the data obtained with values used in various exposure assessment tools for the product types dishwashing liquids, paints and lacquers and fillers. 75th percentiles are used for this comparison, as values used in ConsExpo aim at the 75th percentile as well [21]. In general, a good agreement is evident between our experimental values and those used in ConsExpo. For fillers, ConsExpo uses values for frequency and duration, which are below the values found in our study. ECETOC TRA uses more conservative input values for frequency and duration.

**Table 12:** Comparison of values obtained in the current study with input values of modelling tools for consumer exposure

Parameter	This study^a^	ECETOC TRA^b^[27]	ConsExpo^c^
Hand dishwashing liquid			
Frequency (events/day)	0.9	1.0	1.17 [1]
Duration (min)	17.0	60.0	16.0^d^ [1]
Amount (g)	7.0	50.0	7.0 [1]
Paints and lacquers			
Frequency (events/month)	0.5	30.0	0.09^e^ [2]
Duration (min)	139.2	132.0	120.0^e^ [2]
Amount (g)	388.8	1300.0	1000.0–1300.0^f^ [2]
Fillers			
Frequency (events/month)	0.5	30	0.08–0.17^f^ [3]
Duration (min)	45.8	240	15.0–30.0^f^ [3]
Amount (g)	—	1000.0	0.25–0.5^f^ [3]

^a^75th percentile values

^b^Values taken from subcategories “laundry and dishwashing products”, “solvent rich, high solid, water borne paint” and “fillers and putties”

^c^Values used in ConsExpo aim at the 75th percentile [21]

^d^Assumption for dermal exposure duration, for inhalation exposure duration 60 min are assumed

^e^Identical values for three different scenarios in Bremmer and van Engelen [2]

^f^Range of scenario-specific values, see Bremmer and van Engelen [2] for paints and ter Burg et al. [3] for fillers

In conclusion, for most product types and parameters the methods applied yielded meaningful results. Although this feasibility study did not aim at achieving representativeness, study participants can be considered a reasonable match of the general German population (Table 1). Note that all product types required detailed considerations on specific use conditions for drawing up questionnaires and protocols. For example, prior research was necessary to understand that cockpit spray is marketed both in spray cans and in trigger spray. This information allowed designing questions accordingly.

Retrospective questionnaire approaches (either online or via telephone) allowed retrieving many relevant information: on use frequency, location of application, and conditions of use (e.g. use of gloves, compliance with safety instructions or brand loyalty). Protocols are more favourable for measuring information on a continuous scale, such as use duration and amount of product used. Protocols might also be useful to give more precise figures for room sizes, although this approach would need to provide detailed instructions to participants on how to estimate or measure the dimensions of a room.

In the feasibility study, large groups of product types were assessed together in the case of paints and lacquers and shoes made of rubber or plastic. Grouping similar product types together allows for covering a larger number of product types in one step. But care is required to ascertain that meaningful results are obtained. In the case of paints,use situations varied from painting small hobby items indoors to large arbours or sheds outdoors. If a group approach is chosen, sufficient information needs to be retrieved on use conditions to differentiate exposure scenarios and larger numbers of participants are required to achieve a sufficiently high number of answers when stratifying according to these exposure scenarios

For articles, retrospective questionnaire approaches proved to be the method of choice, if tasks such as weighing products are to be incorporated. This way, key information on many article types can be obtained in a resource-efficient manner.

As shown above for the correlation between amounts and use duration, individual sets of values for different parameters obtained with the same method allow for scrutinising whether parameters are depending of each other. Knowledge about parameter dependency is important for interpreting the outcome of exposure estimates when using these values. Therefore, correlations between parameters should be checked in future surveys.

### Proposal for a large consumer survey

The feasibility study showed that the methods applied worked well and provided valid data for the parameters investigated, which could immediately be used in exposure assessments. For product types consisting of mixtures, the most promising methods are a combination of retrospective questionnaire surveys with protocol methods requiring participants to measure and document values on their own. For articles, questionnaire surveys, which include weighing tasks, are sufficient to retrieve relevant use information.

It is expected that the same methods can be applied to the many product types in use by consumers. However, product-type-specific considerations are still required for designing adequate surveys. For example, for dishwashing liquids it is important to ask for other uses such as cleaning purposes to capture all relevant exposure situations.

The experiences from the feasibility study were used to design the outline of a larger survey, with the capability of covering approximately 24 product types per year. A detailed description of the proposed study design can be found in a parallel publication (Recke et al., 2018, in preparation).

## Conclusions

The feasibility study presented here showed that existing data gaps regarding consumer behaviour data required for exposure assessment can be closed by performing well-planned consumer surveys. Relevant information was obtained for all parameters (frequency, duration, amounts, location of use and other use conditions) and product types investigated. It is concluded that a panel-based combination of questionnaire and protocol methods is a resource-efficient way to perform large surveys covering many different types of consumer products.

## Electronic supplementary material

Supplemental Information

SI 1 Chemical Frequency Questionnaire on all six examined products

SI 2 Recall Foresight Questionnaire on all six examined products

SI 3 Protocol cockpit spray

SI 4 Protocol cockpit spray with camera

SI 5 Protocol dishwashing detergent

SI 6 Protocol dishwashing detergent with camera

SI 7 Protocol filler

SI 8 Protocol filler with camera

SI 9 Protocol paints and lacquers

SI 10 Protocol paints and lacquers with camera
