# Supplementary material for: Consumer behaviour survey for assessing exposure from consumer products: a feasibility study
Source: J Expo Sci Environ Epidemiol. 2018 May 23;29(1):83–94. doi: 10.1038/s41370-018-0040-2 (PMC6760613; doi:10.1038/s41370-018-0040-2)
Supplement: Supplementary file 1 — Supplemental Information [file 41370_2018_40_MOESM1_ESM.docx]

**Supplemental Information to Schneider et al. 2018**

**Consumer behaviour survey for assessing exposure from consumer products: a feasibility study**

**Note:**

Attached are questionnaires and protocols used in the consumer survey. Please note that original documents were provided to survey participants in German language and were translated in order to make it available as supplemental information.

SI 1 Chemical Frequency Questionnaire on all six examined products

SI 2 Recall Foresight Questionnaire on all six examined products

SI 3 Protocol cockpit spray

SI 4 Protocol cockpit spray with camera

SI 5 Protocol dishwashing detergent

SI 6 Protocol dishwashing detergent with camera

SI 7 Protocol filler

SI 8 Protocol filler with camera

SI 9 Protocol paints and lacquers

SI 10 Protocol paints and lacquers with camera
