# Supplementary material for: Consumer behaviour survey for assessing exposure from consumer products: a feasibility study
Source: J Expo Sci Environ Epidemiol. 2018 May 23;29(1):83–94. doi: 10.1038/s41370-018-0040-2 (PMC6760613; doi:10.1038/s41370-018-0040-2)
Supplement: Supplementary file 2 — SI 1 Chemical Frequency Questionnaire on all six examined products [file 41370_2018_40_MOESM2_ESM.docx]

**Chemical Frequency Questionnaire - Chemicals in everyday life**

1. **First of all, we would like to know how often you have used the following products on average in the last 12 months. Please keep in mind that some things are used more frequently and some less frequently in the course of the year. We are interested in the average use. And we are only interested in the private use. That means: If you use something professional, it is not of importance here.**

*Scale: not at all, Less than once a month, 1-3 times per month, once a week, 2-3 times per week, 4-5 times per week, 6-7 times per week*

1. How often have you used hand dishwashing liquid for private purposes in the last 12 months?
2. How often have you used cockpit spray for private purposes in the last 12 months, i. e. a cleaning agent for spraying for the interior of the car? (Other things like cleaning-wipes are not of interest here.)
3. How often have you used lacquers or craft, hobby or artist paint to paint small to large objects or for drawing / painting in the last 12 months for private purposes? (Color for painting walls are not of interest here.)
4. How often have you used powdered fillers, which have to be mixed with water, for the repair of walls and floors, for filling joints, etc. in the last 12 months for private purposes? (Finished filler is not of interest here.)
5. In the last 12 months, have you been wearing shoes that are predominantly made of plastic or rubber? By this we mean for example flip-flops, crocs, sandals and similar shoes made of plastic or rubber. (Possibly add in the cold season: Please keep in mind that some of these shoes are only worn in summer.) (Rubber boots are not of interest here.) yes/no
6. And how often have you used writing utensils such as ball-pens, pencils, crayons or similar in the last 12 months? This time we are interested in the private as well as the professional use.

Control questions (except for 1e) to ensure once again that respondents have understood the scale correctly. In addition, for answer “1-3 times per month” it must be clarified, whether the product is used up to 15 days a year or more.

| *Less than once a month* | So when you add it all up, you have used ……. less than 12 days in the past 12 months. Is that correct? |
| --- | --- |
| *1-3 times per month* | So when you add it all up, have you used …….. up to 15 times or more than 15 times in the last 12 months? |
| *once a week* | So when you add it all up, you have used ……. on at least 52 days in the past 12 months. Is that correct? |
| *2-3 times per week* | So when you add it all up, you have used ……. on at least 104 days in the past 12 months. Is that correct? |
| *4-5 times per week* | So when you add it all up, you have used ……. on at least 208 days in the past 12 months. Is that correct? |
| *6-7 times per week* | So when you add it all up, you have used ……. on at least 312 days in the past 12 months. Is that correct? |

Hand dishwashing liquids

*If hand dishwashing liquids were used in the last 12 months.*

Now we would like to know more about how and where you use hand dishwashing liquids:

1. **You just said that you used hand dishwashing liquids about [insertion frequency from question 1] in the last year. Just think about the days when you washed the dishes: Did you usually do that once or several times a day using hand dishwashing liquids?**
2. **If several times: And how often do you typically wash the dishes with hand dishwashing liquid on such a day?**

……… times

1. **In which room do you usually use hand dishwashing liquids?***(Int.: Please do not read out, assign answers.)*

- kitchen
- bathroom
- other room: ……………………………….

1. **Do you usually wear gloves when washing the dishes with hand dishwashing liquids?**

- yes, always
- sometimes
- no, never

1. **Do you use hand dishwashing liquids for other purposes?**

- yes
- no

**If yes: 6a) What else do you use the hand dishwashing liquids for?** *(Int.: Do not read out, assign answers.)*

- for washing hands
- for cleaning surfaces
- for something else: …………………………………………………….

**If yes: 6b) And do you dilute the hand dishwashing liquid with water, when using it for
< insertion answer from 6a>?**

- yes
- no

1. **Do you always use the same brand of hand dishwashing liquid or change every now and then?**

- I always use the same brand. 🡪 7a) Which one do you use? Can you please tell me the brand?
- I switch between different brands.

Shoes made of plastic or rubber

*If shoes made of plastic or rubber worn in the past 12 months.*

Let's talk about the shoes made of plastic or rubber.

1. **What plastic or rubber shoes have you worn in the last 12 months? Multiple answers are possible.**

- thong sandals
- flip-flops
- bathing shoes
- sandals
- Crocs
- Other shoes: ………………………………………………
- rubber boots (only for control purposes, if only rubber boots are worn, question 1 must be changed to "not at all")

*In case of multiple answers, ask the following questions with respect to the shoe with the largest surface.*

1. **If several shoes were mentioned: In which of these shoes do your feet have contact with the largest surface of plastic or rubber? Please take into account the sole as well as the top of the shoes.**

*(Only the shoes indicated in question 8 are displayed, except rubber boots)*

- thong sandals
- flip-flops
- bathing shoes
- sandals
- Crocs
- Other shoes: ………………………………………………

Now we would like to learn more about your <insert the shoe with the largest surface>.

1. **How often have you worn these <insert the shoe with the largest surface> in the last 12 months? Please keep in mind that some plastic shoes may also have seasonal differences in usage, so you wear these shoes more often in some months than in others. We would like to know the average usage over the last 12 months.**

- less than once a month
- 1-3 times per month
- once a week
- 2-3 times per week
- 4-5 times per week
- 6-7 times per week

1. ***Control question, to ensure once again that respondents have understood the scale correctly. In addition, for answer 1-3 times per month must be clarified, if the product is used up to 15 days a year or more.***

| *Less than once a month* | So when you add it all up, you have used ……. less than 12 days in the past 12 months. Is that correct? |
| --- | --- |
| *1-3 times per month* | So when you add it all up, have you used ……. up to 15 times or more in the last 12 months? |
| *once a week* | So when add it all up, you have used ……. in the past 12 months on at least 52 days. Is that correct? |
| *2-3 times per week* | So when you add it all up, you have used ……. in the past 12 months on at least 104 days. Is that correct? |
| *4-5 times per week* | So when you add it all up, you have used ……. in the past 12 months on at least 208 days. Is that correct? |
| *6-7 times per week* | So when you add it all up, you have used ……. in the past 12 months on at least 312 days. Is that correct? |

1. **And do you wear these <insert the shoe with the largest surface> evenly throughout the year or are there seasonal variations?**

- Wear them year-round
- Wear them seasonally differently

***If 12=seasonally differently 🡪 question 12a:* And in which months or at what seasons do you wear these shoes more often than in others?** …………………………. (note month or season)

1. **When you wear these <insert the shoe with the largest surface>, how many hours a day do you usually do that?**

- Less than 1 hour
- 1 up to 2 hours
- 2 up to 4 hours
- 4 up to 6 hours
- 6 up to 8 hours
- 8 up to 10 hours
- 10 up to 12 hours
- 12 up to 14 hours
- more than 14 hours

1. **What material are the <insert the shoe with the largest surface> exactly made of?**

- plastic
- rubber
- other, in fact: ……………………………………
- material mix: ……………………………………

1. **Are these <insert the shoe with the largest surface> lined or have fabric inside?**

- yes
- no

1. **Do you usually wear socks in your <insert the shoe with the largest surface>?**

- yes
- sometimes
- no

Cockpitspray

*If cockpit spray used in the last 12 months.*

Now let's get to the cockpit spray for the interior cleaning of the car.

1. **Do you usually use cockpit spray from a spray can or a pump spray? (Explanation: A spray can is a metal can that is set under pressure. Content is coming out of the container as long as the spray button is pressed. With a pump spray you first have to build pressure in the container (often made of glass or plastic). This is done by pressing the spray button or a lever / handle. Then only a certain amount will come out of the container per handle operation, no matter how long you press it.)**

- Spray can
- Pump spray
- Something else: ……………………………….. (control question, possibly filter out or change question 1 to “not at all”)

1. **When cleaning your car with cockpit spray, how much time does it usually take, including spraying and wiping?**

- less than 15 minutes
- approx. 15 minutes
- approx. 30 minutes
- approx. 45 minutes
- approx. 1 hour
- approx. 1 hour and 15 minutes
- approx. 1,5 hours
- approx. 1 hour and 45 minutes
- approx. 2 hours
- more than 2 hours, exactly: ………………………………..

1. **And how long do you usually stay in the car after cleaning with the cockpit spray?**

- less than 15 minutes
- approx. 15 minutes
- approx. 30 minutes
- approx. 45 minutes
- approx. 1 hour
- approx. 1 hour and 15 minutes
- approx. 1,5 hours
- approx. 1 hour and 45 minutes
- approx. 2 hours
- more than 2 hours, exactly: ………………………………..

1. **Where does your car usually stand when you clean it with cockpit spray?**

- outdoors
- in a garage
- in a carport
- in another place: ……………………………

1. **Are the doors of your car usually open or closed while cleaning?**

- open *(skip next question)*
- closed

1. **What about the car’s windows? Are these usually open or closed during the interior cleaning with cockpit spray?**

- open
- closed

1. **If you use cockpit spray, do you usually wear gloves or not?**

- yes, always
- sometimes
- no, never

1. **On the container or the packaging of the cockpit spray are application instructions. Do you usually read them before use or do you usually not read them?**

- I usually read them.
- I usually do not read them.

1. **And do you usually follow the instructions given on the cockpit spray or do you usually ignore them? (If usually not read: Even if you did not read the instructions for use last time, are there any general instructions that you follow when using cockpit spray?)**

- I usually follow them. 🡪 Which of the instructions are you following?
- I usually do not follow them.

1. **Do you usually use the same brand of cockpit spray or change it every now and then?**

- I usually use the same brand of cockpit spray. 🡪 Which one? ……………………..
- I switch between different brands of cockpit spray.

Filler

*If fillers used in the last 12 months.*

Let's return to the powdered filler, which is mixed with water for the repair of walls and floors, for the filling of gaps, etc.

1. **If you do something with filler, how much time does that usually take?**

- less than 15 minutes
- approx. 15 minutes
- approx. 30 minutes
- approx. 45 minutes
- approx. 1 hour
- approx. 1 hour and 15 minutes
- approx. 1,5 hours
- approx. 1 hour and 45 minutes
- approx. 2 hours
- more than 2 hours, exactly: ………………………………..

1. **Where do you usually use filler?**

- inside 🡪 In which room(s) exactly: living room / bedroom / children's room / study / cellar / kitchen / bathroom / toilet / hall / elsewhere, namely: ………) *multiple choices possible*
- outdoors

1. **If you use filler, do you usually wear gloves?**

- yes
- sometimes
- no

1. **On the container or packaging of the filler instructions for use can be found. Do you usually read them before use or do you usually not read them?**

- I usually read them.
- I usually do not read them.

1. **And do you usually follow the instructions given on the filler or do you usually ignore them? (If usually not read: Even if you did not read the instructions for use last time, are there any general instructions for use that you keep in mind when using filler?)**

- I usually follow them. 🡪 Which of the instructions are you following?
- I usually do not follow them.

1. **Do you usually use aids such as a scraper or a smoothing trowel for applying the filler?**

- yes: ………………………. *(maybe name some: scraper, smoothing trowel)*
- no

1. **Do you always use the same brand of filler or change it every now and then?**

- I always use the same brand of filler: ……………
- I switch between different brands.

Lacquers and paints

*If lacquers and/or paints used in the past 12 months.*

1. **Which was the last item you painted with paint or lacquer? If you have painted a picture, this is also of interest.**

- Painted item: ………………………..
- I painted a picture.
- Something else: ………………………..
- I painted a wall. *(control question!)*

1. **We stick to this last application: Where exactly did you paint the item: was it inside or outside?**

- inside 🡪 question 33a: And in which room exactly: (living room / bedroom / children's room / study / cellar / kitchen / bathroom / toilet / hall / elsewhere, namely: …………)
- outside

1. **And how much time did the application take in total?**

- less than 15 minutes
- approx. 15 minutes
- approx. 30 minutes
- approx. 45 minutes
- approx. 1 hour
- approx. 1 hour and 15 minutes
- approx. 1,5 hours
- approx. 1 hour and 45 minutes
- approx. 2 hours
- more than 2 hours, exactly: ………………………………..

1. ***If application took place indoors:* And how long have you been in the same room after painting?**

- less than 15 minutes
- approx. 15 minutes
- approx. 30 minutes
- approx. 45 minutes
- approx. 1 hour
- approx. 1 hour and 15 minutes
- approx. 1,5 hours
- approx. 1 hour and 45 minutes
- approx. 2 hours
- more than 2 hours, exactly: ………………………………..

1. **And did you wear gloves when painting?**

- yes
- no

1. **And did you wear other protective clothing when painting?**

- yes: ……………………………………………
- no

1. **On the container or the packaging of paints and lacquers instructions for use can be found. Did you read them before the last application or not?**

- Yes, I read them.
- No, I did not read them.

1. **And did you follow the instructions given when you last used paints and lacquers? (If usually not read: Even if you did not read the instructions for use last time, are there any general instructions for use that you keep in mind when using paints and lacquers?)**

- yes 🡪 Which of the instructions did you follow?
- no

1. **Do you usually use the same brand of paint/lacquer or change it now and then?**

- I always use the same brand of paint/lacquer: ………………….
- I switch between different paints/lacquers.

Pens

*If pens used in the last 12 months.*

1. **What material is the pen you use the most made of? If you use several pens equally often, please tell me the material which the majority of these pens are made of.***(only single choice possible)*

- plastic
- wood
- metal
- Something else: ………………………………….

1. **In which room do you usually use this pen the most?**

- in the office / workplace
- study
- living room
- nursery
- bedroom
- kitchen
- bathroom
- elsewhere: …………………………………

1. **Please estimate how many minutes you hold a pen on a normal day.**

*(Int.: Helpful questions, if the question is hard to be answered: Do you use pens in your work? 🡪 What do you use them for? 🡪 How many times a day do you do that?🡪 How long does one of these processes take? 🡪 roughly sum up*

*Do you use pens in your spare time? 🡪 What do you use them for? 🡪 How many times a day do you do that?🡪 How long does one of these processes take? 🡪 roughly sum up*

*These values are not recorded, but the interviewer should roughly calculate.)*

1. **Do you occasionally chew on the pen or do you intentionally or unconsciously lead the pen to your mouth?**

- yes
- no (skip next question)

1. **You just said that you use a pencil for about ... minutes on a normal day. And what do you estimate: how many minutes do you chew on the pen or deliberately or unconsciously lead it to your mouth?**
2. **We are also interested in the weight of the pen you use the most to estimate the ingredients. Is it possible that you weigh the pen you use the most on a kitchen scale or something similar? If the pen is not tangible because it's in the office, for example, please weigh the pen you use the most at home.**

- Yes, the weight of the most commonly used pen is: ……………. g
- Yes, the weight of a spare pen is: ……………. g
- No, I have no scales at home. / No, I do not want to weigh the pen.

*If shoes made of plastic or rubber worn in the past 12 months*:

1. **Earlier we talked about the <insert the shoe with the largest surface> you wear now and then. For the estimation of the ingredients of the shoes we are also interested in the weight of these shoes. Could you also weigh the shoes right now? Please only weigh a single shoe!**

- Yes, the weight is: ……………. g
- No, the shoes are not at hand. / I do not have scales at home. / I do not want to weigh the shoes.

Statistics

Finally, we have some statistical questions.

**S1. Gender of respondent**

- male
- female

**S2A. In which year were you born?**

**S2B. And in which month?**

**S2C. *(if information about year of birth refused)* Then I will tell you different age groups. Would you please fit into one of them?**

- 18-30 years
- 31-40 years
- 41-50 years
- 51-60 years
- 61-70 years
- 71 years and older

**S4. Which is the highest general education level you have?**

- no qualification yet / still going to school
- Finished school without qualifications
- “Hauptschulabschluss” (elementary school) / Polytechnic secondary school of the GDR with completion of 8th or 9th grade
- „Realschulabschluss“ (Secondary school / high school) / Polytechnic high school of the GDR with completion of 10th grade
- „Fachhochschulreife“, completion of a „Fachoberschule“
- General or subject-related university entrance qualification / “Abitur“ (“Gymnasium” or extended secondary school (EOS), also EOS with vocational training)
- “Abitur” made via adult-education (e. g. evening school)
- Another degree: ………………………
- Refused

**S5. Are you currently working?**

- yes, full-time
- yes, part-time, hourly / occasionally
- no, temporarily not working / unemployed
- no, no longer working / pension
- housewife / houseman
- still in education / student, apprentice etc.
- something else
- refused

**S6. How many people live in your household, including yourself? All persons who live and work together here belong to this household. Please also remember all the children living in the household.**

**S7A. How many of these are 18 years and older?**

**S7B. How many of these are younger than 14 years?**

**S7C. How many of these are between 14 and 17 years old?**

**P1. We now have finished the survey. Thank you for your participation. In the next 3 months, we would like to take a closer look at product safety. This means that we would like to contact you again for about 2 more short interviews. These brief surveys are about the usage of different products in the previous month. In addition, some participants will be asked to take measurements. (This includes the documentation of the quantities used or the duration of the application.) By participating in the study, you make an important contribution to the continuous improvement of consumer protection and product safety. This benefits all consumers. We would be glad, if we could contact you soon two more times. Is that possible?**

**How would you like to be contacted by us? Shall we call again or rather send you an e-mail?**
