# Supplementary material for: Consumer behaviour survey for assessing exposure from consumer products: a feasibility study
Source: J Expo Sci Environ Epidemiol. 2018 May 23;29(1):83–94. doi: 10.1038/s41370-018-0040-2 (PMC6760613; doi:10.1038/s41370-018-0040-2)
Supplement: Supplementary file 3 — SI 2 Recall Foresight Questionnaire on all six examined products [file 41370_2018_40_MOESM3_ESM.docx]

| Before using paints/lacquers |
| --- |
|  |
| **Please write down today's date: __ __. __ __. 2017** |
|  |
| **Which paint or lacquer will you use today?** Please write down the exact brand name that is written on the container. Please note the full name, including any variant names, fragrance information or the like.  🖉 ……………………….……………………….…………………………………………………….  ……………………….……………………….……………………………………………………. |
|  |
| **Do you usually use the same paint or lacquer or do you change the brand now and then?**   - I always use the same brand. - I switch between different brands |
|  |
| **Please weigh the container of the paint or lacquer you want to use right now and enter the displayed weight here. If you want to use several paints / lacquers please weigh all containers together and enter the number of paints / lacquers below.**  If possible, use a balance that measures the weight to one gram. Please make sure that the balance shows "0 grams" before the measurement.  Number of paints/lacquers: 🖉……………………….  Weight before application : 🖉………………………. g |
|  |

| **Please put on the camera now, switch it on and make sure that the camera is running.** |
| --- |
| **We need an accurate idea of the item you want to treat with paint or lacquer. Please try to record this item now as completely as possible with the camera.** It is also helpful if you place a ruler or tape measure on an edge of the object so that it measures its length or height. From this we can later deduce the other dimensions of the object. If you want to treat a two-dimensional surface, then please do the same, but of course we only need one view of the surface. |
|  |
| **Now we need pictures of the paint or lacquer that you will use. Please turn the container in front of the camera if possible at a distance where one can read the most important information.** |
|  |
| **Please start using the paint or lacquer. Once again as a reminder: please proceed as you would without this protocol and the camera.** |

| After using paints/lacquers |
| --- |
|  |
| **Please weigh again the container of the paint or lacquer you have used and enter the displayed weight here. If you have used several paints / lacquers please weigh all containers together and enter the number of paints / lacquers below.** Please make sure that the balance shows "0 grams" before the measurement.  Number of paints/lacquers: 🖉……………………….  Weight after application : 🖉………………………. g |
|  |
| **Where did you use the paint/lacquer today?**   - Outside - Indoor 🡪 in which room exactly? 🖉……………………….……………………….   🡪 How big is this room? 🖉……………………….……………sq. m. |
|  |

| **In case, the application happened indoor: How long have you been in that room after you finished painting?**  🖉 ………………………. minutes | | | | | |
| --- | --- | --- | --- | --- | --- |
|  | | | | | |
| **In case, the application took place indoor: Were the windows open or closed during application of the paint?**   - Windows open ⬜ Windows closed | | | | | |
|  | | | | | |
| **In case, the application took place indoor: Were the doors open or closed during application of the paint?**   - Doors open ⬜ Doors closed | | | | | |
|  | | | | | |
| **Did you wear gloves while painting or not?**   - Yes, I wore gloves ⬜ No, I did not wear gloves | | | | | |
|  | | | | | |
| **Did you wear other protective clothing during painting or not?**   - Yes 🡪 What exactly? 🖉 ……………………….……………………….………………………. - No | | | | | |
|  | | | | | |
| **On the container or the packaging of the paint or lacquer you can find instructions for use. Did you read them today?**   - Yes, I read them. ⬜ No, I did not read them. | | | | | |
|  | | | | | |
| **Did you follow the instructions for use on the container today?** (Even if you did not read these instructions this time, it is possible that you know them from previous applications.)   - Followed instructions🡪 Which instruction did you follow?   🖉 ……………………….……………………….………………………………………  ……………………….……………………….………………………………………  ……………………….……………………….………………………………………   - I did not follow the instructions. | | | | | |
|  | | | | | |
| **Please rate the completion of the protocol briefly. Just mark the corresponding number.** | | | | | |
| How interesting was the completion of the protocol on a scale from 1 = "very interesting" to 5 = "not at all interesting" for you? | 1 | 2 | 3 | 4 | 5 |
|  | | | | | |
| How do you rate the length of the protocol on a scale from 1 = "was too long" to 5 = "was too short"? | 1 | 2 | 3 | 4 | 5 |
|  | | | | | |
| How do you rate the comprehensibility of the questions on a scale from 1 = "were understandable" to 5 = "were incomprehensible"? | 1 | 2 | 3 | 4 | 5 |
|  | | | | | |
| How much fun did you have on a scale from 1 = "was fun" to 5 = "was not fun"? | 1 | 2 | 3 | 4 | 5 |
|  | | | | | |
| How elaborate was the participation on a scale of 1 = “not at all complex" to 5 =" very complex"? | 1 | 2 | 3 | 4 | 5 |
|  | | | | | |
| Would you participate in the survey 1 = “again" to 5 = "not participate again"? | 1 | 2 | 3 | 4 | 5 |
| Here is space for further comments / notes to us. | | | | | |

**Thank you for your cooperation!**

Please return the filled-in protocol and the camera to us immediately in the package that we have sent to you. You can use the stamped sticker which we have sent to you.
