# Supplementary material for: Consumer behaviour survey for assessing exposure from consumer products: a feasibility study
Source: J Expo Sci Environ Epidemiol. 2018 May 23;29(1):83–94. doi: 10.1038/s41370-018-0040-2 (PMC6760613; doi:10.1038/s41370-018-0040-2)
Supplement: Supplementary file 4 — SI 3 Protocol cockpit spray [file 41370_2018_40_MOESM4_ESM.docx]

**Recall Foresight Questionnaire - Chemicals in everyday life**

1. **First of all, we would like to know how often you have used the following products in the last 4 weeks. We are only interested private use. If you use something professional, that is not of importance here.**

*Scale: not at all, less than once a week, 1-3 times per week, 4-5 times per week, 6-7 times per week*

1. How often have you used hand dishwashing liquid for private purposes in the last 4 weeks?
2. How often have you used cockpit spray for private purposes in the last 4 weeks, i. e. a cleaning agent for spraying for the interior of the car? (Other things like cleaning-wipes are not of interest here.)
3. How often have you used lacquers or craft, hobby or artist paint to paint small to large objects or for drawing / painting in the last 4 weeks for private purposes? (Color for painting walls are not of interest here.)
4. How often have you used powdered fillers, which have to be mixed with water, for the repair of walls and floors, for filling joints, etc. in the last 4 weeks for private purposes? (Finished filler is not of interest here.)
5. In the last 4 weeks, have you been wearing shoes that are predominantly made of plastic or rubber? By this we mean for example flip-flops, crocs, sandals and similar shoes made of plastic or rubber. (Possibly add in the cold season: Please keep in mind that some of these shoes are only worn in summer.) (Rubber boots are not of interest here.) yes/no
6. And how often have you used writing utensils such as ball-pens, pencils, crayons or similar in the last 4 weeks? This time we are interested in the private as well as the professional use.

Hand dishwashing liquids

*If hand dishwashing liquids used in the last 4 weeks.*

1. **Now we would like to know a bit more exactly when you last used the dishwashing liquid. About how many days ago was that?**

- That was today.
- That's ......... days ago.
- I do not know.

1. ***If previous question = I don´t know:* Based on the answers below, can you please estimate, when you last used hand dishwashing detergent within the last 4 weeks? Was that …**

- today
- within the last 7 days
- 8 to 14 days ago
- 15 to 21 days ago
- 22 to 31 days ago
- more than 31 days ago *(Control answer: skip block and Q1 dishwashing liquid = “not at all”)*

1. **And how often have you washed the dishes on the day you last used hand dishwashing liquid?**

……… times

1. **In which room did you use the hand dishwashing liquid when you last washed the dishes?** *(Int.: Please do not read out, assign answers)*

- kitchen
- bathroom
- another room: ……………………………….

1. **Did you wear gloves the last time you were washing the dishes?**

- yes
- no

1. **Did you also use hand dishwashing detergent for other purposes in the last four weeks?**

- yes
- no

**if yes: 7a) What did you last use it for?** *(Int .: Do not read out, but assign answers.)*

- for washing hands
- for cleaning surfaces
- for something else: …………………………………………………….

**Falls ja: 6b) And did you dilute the hand dishwashing liquid with water the last time you used it for
<insertion answer from 6a>?**

- yes
- no

1. **Which brand of hand washing liquid did you use the last time you washed the dishes?**

- ……………………..

1. **Do you always use the same brand of hand dishwashing liquid or change it every now and then?**

- I always use the same brand.
- I switch between different brands.

Shoes made of plastic or rubber

*If shoes made of plastic or rubber worn in the last 4 weeks.*

Let's talk about your shoes made of plastic or rubber.

1. **What kinds of plastic or rubber shoes have you worn in the last 4 weeks? Several entries are possible.**

- thong sandals
- flip-flops
- bathing shoes
- sandals
- Crocs
- Other shoes: ………………………………………………
- (do not read out) rubber boots (only for control purposes, if only rubber boots are worn, question 1 must be changed to "not at all"))

1. **If several shoes were mentioned: In which of these shoes do your feet have contact with the largest surface of plastic or rubber? Please take into account the sole as well as the top of the shoes.** *(Only the shoes indicated in question 10 are displayed, except rubber boots.)*

- thong sandals
- flip-flops
- bathing shoes
- sandals
- Crocs
- Other shoes: ………………………………………………

1. **What material are the <insert from question 11> exactly made of? (Possibly: If you are not sure, it would be very nice of you if you take a quick look.)**

- plastic
- rubber
- other, in fact: ……………………………………
- material mix: …………………………………………….

1. **We stick with this <insert from question 11>. Did you wear these <insert from question 11> with or without socks the last time?**

- with socks
- without socks

1. **How often have you worn these <insert from question 11> in the last 4 weeks?** (open answer, number between 1 and 31 times)
2. **And when did you wear the <insert from question 11> the last time? About how many days ago was that?** (Filter: if question 14 = 31 times)

- That was today.
- That's ......... days ago.
- I don´t know.

1. **If previous question = I don´t know: Based on the answers below, could you please estimate, when you wore the <insert from question 11> the last time within the last four weeks? Was that ….**

- today
- within the last 7 days
- 8 to 14 days ago
- 15 to 21 days ago
- 22 to 31 days ago
- more than 31 days ago (control answer: skip block and Q1 shoes = “no”)

1. **We are also interested in the weight of the shoes to estimate the ingredients. Is it possible that you briefly weigh the shoes on a kitchen scale or similar? Please only weigh a single shoe!**

- Yes, the weight is: ………..
- No, the shoes are not at hand.
- No, I have no scales at home.
- No, I do not want to weigh the shoes.
- The shoes were weighed the last time. *(import data from that wave)*

Cockpit spray

*If cockpit spray used in the last 4 weeks.*

Let's get to the cockpit spray for the interior cleaning of the car.

1. **When did you use the cockpit spray the last time within the last 4 weeks? How many days ago was that about?**

- That was today.
- That's ......... days ago.
- I don´t know.

1. **If previous question = I don´t know: Based on the answers below, could you please estimate, when you last used the cockpit spray in the last 4 weeks? Was that ….**

- today
- within the last 7 days
- 8 to 14 days ago
- 15 to 21 days ago
- 22 to 31 days ago
- more than 31 days ago (Control, skip block and Q1 cockpit spray = not at all)

1. **Did you use cockpit spray from a spray can or a pump spray the last time?** **(Explanation: A spray can is a metal can that is set under pressure. Content is coming out of the container as long as the spray button is pressed. With a pump spray you first have to build pressure in the container (often made of glass or plastic). This is done by pressing the spray button or a lever / handle. Then only a certain amount will come out of the container per handle operation, no matter how long you press it.)**

- spray can
- pump spray
- I don´t know

1. **What is your estimate, how long did it take you to clean the car with cockpit spray including spraying and wiping the last time? Was that …**

- less than 15 minutes
- approx. 15 minutes
- approx. 30 minutes
- approx. 45 minutes
- approx. 1 hour
- approx. 1 hour and 15 minutes
- approx. 1,5 hours
- approx. 1 hour and 45 minutes
- approx. 2 hours
- more than 2 hours, exactly: ………………………………..

1. **And how long did you stay in the car immediately after cleaning it with the cockpit spray the last time? Was that….**

- less than 15 minutes
- approx. 15 minutes
- approx. 30 minutes
- approx. 45 minutes
- approx. 1 hour
- approx. 1 hour and 15 minutes
- approx. 1,5 hours
- approx. 1 hour and 45 minutes
- approx. 2 hours
- more than 2 hours, exactly: ………………………………..

1. **What do you think: How many sprays did you make today during car care and how many seconds did one of these sprays last?**

Number of sprays 🖉___________

Average duration of a spray: ______ seconds

1. **Where was your car when you last cleaned it with cockpit spray?**

- outdoors
- in a garage
- in the carport
- in another place: ……………………………

1. **Were the doors of the car open or closed during the last cleaning?**

- open (skip next question)
- closed

1. **What about the car’s windows? Were they open or closed during the last interior cleaning with cockpit spray?**

- open
- closed

1. **And did you wear gloves the last time you cleaned the interior with cockpit spray or not?**

- yes
- no

1. **On the container or the packaging of the cockpit spray are application instructions. Did you read them before the last use or not?**

- I read them.
- I did not read them.

1. **And have you followed the application notes given on the cockpit spray the last time you used it or not?**

- I followed them. 🡪 Which of the instructions did you follow?
- I did not follow them.

1. **Which brand did you use last time you used cockpit spray?** *(Int.: If necessary, ask the respondent to look it up if he does not know it.)* ……………………..

Filler

*If fillers used in the last 4 weeks.*

Let's return to the powdered filler, which is mixed with water for the repair of walls and floors, for the filling of gaps, etc.

1. **When did you use the powdered filler to mix with water the last time within the last 4 weeks? About how many days ago was that?**

- That was today.
- That's ......... days ago.
- I don´t know.

1. **If previous question = I don´t know: Based on the answers below, could you please estimate, when you used the filler the last time in the last 4 weeks? Was that ….**

- today
- within the last 7 days
- 8 to 14 days ago
- 15 to 21 days ago
- 22 to 31 days ago
- more than 31 days ago (control answer: skip block and Q1 filler = “not at all”)

1. **And for what purpose exactly did you use the filler the last time? What did you fill with it?**

………………………………..

1. **When you last used the filler, how long did it take to mix it with water?**

………………………………..

1. **And about how long did the actual filling take? Was that…**

- less than 15 minutes
- approx. 15 minutes
- approx. 30 minutes
- approx. 45 minutes
- approx. 1 hour
- approx. 1 hour and 15 minutes
- approx. 1,5 hours
- approx. 1 hour and 45 minutes
- approx. 2 hours
- more than 2 hours, exactly:: ………………………………..

1. **And where exactly did you use the filler?**

- inside 🡪 question 37a: In which room(s) exactly: (living room / bedroom / children's room / study / cellar / kitchen / bathroom / toilet / hall / elsewhere, that is: ……….)
- outside

1. **And did you wear gloves?**

- yes
- no

1. **On the container or packaging of the filler instructions for use can be found. Did you read them before last use or not?**

- I read them.
- I did not read them.

1. **And have you followed the instructions for use on the container or packaging the last time you used the filler?**

- I followed them. 🡪 Which of the instructions did you follow?
- I did not follow them.

1. **Did you have an aid the last time you used filler? For example used a scraper or a smoothing trowel for applying the filler?**

- yes: ………………………. (maybe name some: scraper, smoothing trowel)
- no

1. **Which brand of filler did you use exactly? Please tell me the product name. (Int.: Possibly ask if the respondent can get the product and read out the name.)**

………………………………..

1. **And do you always use this brand of filler or change it every now and then?**

- I always use the same filler.
- I switch between different fillers.
- This is my first use of powdered filler.

Lacquers and paints

*If lacquers and/or paints used in the last 4 weeks.*

1. **When did you use paints or lacquers the last time within the last 4 weeks? How many days ago was that about?**

- That was today.
- That's ......... days ago.
- I don´t know.

1. **If previous question = I don´t know: Based on the answers below, could you please estimate when you used the paints or lacquers the last time in the last four weeks? Was that**

- today
- within the last 7 days
- 8 to 14 days ago
- 15 to 21 days ago
- 22 to 31 days ago
- more than 31 days ago (control answer, skip block and Q1 paints and lacquers = “not at all”)

1. **Which was the last item you painted with paint or lacquer? If you have painted a picture, this is also of interest.**

- Painted item: …………………………………..
- I painted a picture.
- Something else: …………………………………..
- I painted a wall. (control answer 🡪 skip block and Q1 paints and lacquers = “not at all”)

1. **We stick to this last application: Where exactly did you paint the item: was it inside or outside?**

- inside 🡪 question 46a: And in which room exactly: (living room / bedroom / children's room / study / cellar / kitchen / bathroom / toilet / hall / elsewhere, that is: …………….)
- outside

1. **And how much time did the application take in total?**

- less than 15 minutes
- approx. 15 minutes
- approx. 30 minutes
- approx. 45 minutes
- approx. 1 hour
- approx. 1 hour and 15 minutes
- approx. 1,5 hours
- approx. 1 hour and 45 minutes
- approx. 2 hours
- more than 2 hours, exactly: ………………………………..

1. **And how long have you been in the same room after painting? (Filter: only if product is used indoors)**

- less than 15 minutes
- approx. 15 minutes
- approx. 30 minutes
- approx. 45 minutes
- approx. 1 hour
- approx. 1 hour and 15 minutes
- approx. 1,5 hours
- approx. 1 hour and 45 minutes
- approx. 2 hours
- more than 2 hours, exactly: ………………………………..

1. **And did you wear gloves when painting?**

- yes
- no

1. **And did you wear other protective clothing when painting?**

- yes: ……………………………………………
- no

1. **On the container or the packaging of paints and lacquers instructions for use can be found. Did you read them before the last application or not?**

- Yes, I read them.
- No, I did not read them.

1. **And did you follow the instructions given when you used paints and lacquers the last time?**

- yes 🡪 Which of the instructions did you follow?
- no

1. **Which brand of paint/lacquer did you use exactly? Please tell me the product name. (Int.: Possibly ask if the respondent can get the product and read out the name.)**

………………………………..

1. **And for this particular purpose (question 45), do you always use the same brand or do you change it every now and then?**

- I always use the same brand.
- I change between different brands.
- This is my first use of paints/lacquers.

Pens

*If pens used in the last 4 weeks.*

Let us now talk about the writing instruments: the pens, pencils, crayons and the like**.**

1. **When did you last use a writing utensil during the last 4 weeks, either for private of professional use? About how many days ago was that?**

- That was today.
- That's ......... days ago.
- I don´t know.

1. **If previous question = I don´t know: Based on the answers below, could you please estimate when you used a writing utensil the last time in the last four weeks? Was that ….**

- today
- within the last 7 days
- 8 to 14 days ago
- 15 to 21 days ago
- 22 to 31 days ago
- more than 31 days ago (Control, skip block and Q1 pens = not at all)

1. **What material is the pen you used the last time made of?** *(Int.: only single choice possible)*

- plastic
- wood
- metal
- Something else: ………………………………….

1. **In which room did you use this pen the last time?**

- in the office / workplace
- study
- living room
- nursery
- bedroom
- kitchen
- bathroom
- somewhere else: …………………………………

1. **Please estimate: For how many minutes did you hold the pen in your hand on this day?***(Int.: Helpful questions, if the question is hard to be answered:*

*Why did you use the pen on this day? 🡪 How long did this process take? 🡪 roughly sum up*

*These values are not recorded, but the interviewer should roughly calculate.)*

1. **And the last time you used the pen, did you chew on it or deliberately or unconsciously lead the pen to the mouth?**

- yes
- no (skip next question)

*(First sentence only if question 60 is not answered with “I don´t know”)*

1. **You just said that you used the pen for ... minutes the last time. And what is your estimate, for how many minutes have you chewed on the pen or deliberately or unconsciously led it to the mouth?**
2. **For the estimation of its ingredients we are also interested in the weight of the pen. Is it possible that you briefly weigh the pen you used the last time on a kitchen scale or similar? If the pen is not tangible because it's in the office for example, please weigh the pen you use the most at home.**

- Yes, the weight of the pen is: ……………. g.
- Yes, the weight of a spare pen is: ……………. g.
- No, have no scales at home.
- No, I do not want to weigh the pen.
- I have already weighed pen. *(import data from last wave)*

1. **Finally, we would like to know how likely you will use the products below within the next 4 weeks.**

*(scale: 1 = no way, 2 = is unlikely, 3 = is likely, 4 = I will definitely use it.)*

- How likely will you use hand dishwashing liquid for private purposes in the next 4 weeks?
- How likely will you use cockpit spray for private purposes in the next 4 weeks, which means you will use a cleaner for the interior of the car for spraying? (Wipes or the like are not of interest here.)
- How likely will you use lacquers or craft, hobby or artist paint to paint small to large objects or to draw / paint for private purposes in the next 4 weeks? (Color for painting walls is not of interest here.)
- How likely will you use powdered filler that is mixed with water for the repair of walls and floors, for the filling of joints etc. for private purposes in the next 4 weeks? (Finished filler is not of interest here.)

Measuring instruments in the household

1. **Which of the following do you possess in your household?**

- tape measure
- ruler
- clock with seconds (also on the mobile phone)
- kitchen scale
- bathroom scale
- letter scale
- other fine scales (up to 500 g)

Selection of the subjects for the special tasks (measurement protocols and camera)

*For the special tasks all respondents are qualified, which will "probably or definitely" use cockpit spray, filler or paint/lacquer in the next 4 weeks.*

As part of our study, we would like to know more about how exactly the population in Germany applies different products. To investigate this, a simple survey on the phone is not enough. Rather, it is necessary to accurately document the application while it is going on or shortly afterwards. Since you said you may use / definitely will use …………….. in the next 4 weeks, we would like to ask you to fill out a short protocol during the application. We are interested in how much paint / filler / cockpit spray you use, how many minutes or hours it will take to use it and how exactly you use the product. We will thank you for your efforts with a 10 € amazon voucher. We would like to send you the protocol by post. You will also receive a stamped envelope, which you can use to send the protocol back to us free of charge. The whole task will not cost you anything that time.

May we send you a protocol?

- yes 🡪 note adress
- no 🡪 Does not want to give the address. 🡪 Alternatively, we can send you the protocol by e-mail. When you have filled it in, send it as a photo by e-mail or print it out and send it to us by post.
- No, I generally do not want to.
