# Supplementary material for: Consumer behaviour survey for assessing exposure from consumer products: a feasibility study
Source: J Expo Sci Environ Epidemiol. 2018 May 23;29(1):83–94. doi: 10.1038/s41370-018-0040-2 (PMC6760613; doi:10.1038/s41370-018-0040-2)
Supplement: Supplementary file 5 — SI 4 Protocol cockpit spray with camera [file 41370_2018_40_MOESM5_ESM.docx]

| Bevor using cockpit spray | |
| --- | --- |
|  | |
| **Please write down today's date: __ __ . __ __. 2017** | |
|  | |
| **Which cockpit spray will you use today?** Please write down the exact brand name that is written on the container. Please note the full name, including any variant names, fragrance information or the like.  🖉 ……………………….……………………….…………………………………………………….  ……………………….……………………….……………………………………………………. | |
|  | |
| **Is this a cockpit spray from the spray can or a pump spray?** A spray can is a metal can that is under pressure. As long as the spray button is pressed, cockpit spray will leave the can. For a pump spray you have to build up pressure in the container (often made of glass or plastic) first. This is done by pressing the spray button or a lever / handle. Then only a certain amount will come out of the container, no matter how long you press it. | |
| **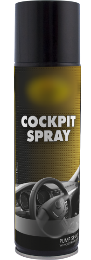**  ⬜ Spray can | 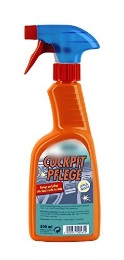  ⬜ Pump spray |
|  | |
| **Please weigh the container of the cockpit spray that you want to use now and enter the displayed weight here.** If possible, use a balance that measures the weight to one gram. Please make sure that the balance shows "0 grams" before the measurement.  Weight before use: 🖉…………………………………. g | |
| **Please put on the camera now, switch it on and make sure that the camera is running.** | |
|  | |
| **First, we need pictures of the cockpit spray you will be using. Please turn the container in front of the camera if possible at a distance where one can read the most important information.** | |
|  | |
| **Please start to clean the interior of the car now. Once again as a reminder: Please proceed in the same way as you would do without this protocol.** | |

| After using the cockpit spray |
| --- |
|  |
| **If you are done with the application of the cockpit spray including spraying and wiping for today, you can turn off the camera.** |
|  |
| **Please weigh the container of the cockpit spray that you have used and enter the displayed weight here.** Please make sure again that the balance shows "0 grams" before the measurement.  Weight after application: 🖉**………………………………….** g |
|  |
| **Where was your car parking when you cleaned it today with cockpit spray?**   - Outdoors - In a garage - In a carport - At a different place: 🖉………………………………………………………………… |

| **Were the doors open or closed during cleaning?**   - Open - Closed |
| --- |
|  |
| **And what about the windows of the car? Were these open or closed during the cleaning process with cockpit spray?**   - Open - Closed |
|  |
| **And did you wear gloves when cleaning the interior with cockpit spray or not?**   - Yes, I wore gloves - No, I did not wear gloves |
|  |
| **And did you wear other protective clothing during the cleaning process with cockpit spray?**   - Yes🡪 What exactly🖉………………………………………………………………………………… - No |
|  |
| **On the container or the packaging of the cockpit spray you can find instructions for use. Did you read them today?**   - Yes, I read them. - No, I did not read them. |
|  |

| **Did you follow the instructions for use on the container today?**   - Followed instructions🡪 Which instruction did you follow?   🖉 ……………………….……………………….………………………………………  ……………………….……………………….………………………………………  ……………………….……………………….………………………………………   - I did not follow the instructions. | | | | | |
| --- | --- | --- | --- | --- | --- |
|  | | | | | |
| **Please rate the completion of the protocol briefly. Just mark the corresponding number.** | | | | | |
| How interesting was the completion of the protocol on a scale from 1 = "very interesting" to 5 = "not at all interesting" for you? | 1 | 2 | 3 | 4 | 5 |
|  | | | | | |
| How do you rate the length of the protocol on a scale from 1 = "was too long" to 5 = "was too short"? | 1 | 2 | 3 | 4 | 5 |
|  | | | | | |
| How do you rate the comprehensibility of the questions on a scale from 1 = "were understandable" to 5 = "were incomprehensible"? | 1 | 2 | 3 | 4 | 5 |
|  | | | | | |
| How much fun did you have on a scale from 1 = "was fun" to 5 = "was not fun"? | 1 | 2 | 3 | 4 | 5 |
|  | | | | | |
| How elaborate was the participation on a scale of 1 = “not at all complex" to 5 =" very complex"? | 1 | 2 | 3 | 4 | 5 |
|  | | | | | |
| Would you participate in the survey 1 = “again" to 5 = "not participate again"? | 1 | 2 | 3 | 4 | 5 |
| Here is space for further comments / notes to us. | | | | | |

**Thank you for your cooperation!**

Please return the filled-in protocol and the camera to us immediately in the package that we have sent you. You can use the stamped sticker which we have sent to you.
