# Supplementary material for: Consumer behaviour survey for assessing exposure from consumer products: a feasibility study
Source: J Expo Sci Environ Epidemiol. 2018 May 23;29(1):83–94. doi: 10.1038/s41370-018-0040-2 (PMC6760613; doi:10.1038/s41370-018-0040-2)
Supplement: Supplementary file 6 — SI 5 Protocol dishwashing detergent [file 41370_2018_40_MOESM6_ESM.docx]

| Bevor using cockpit spray | |
| --- | --- |
|  | |
| **Please write down today's date: __ __ . __ __. 2017** | |
|  | |
| **Which cockpit spray will you use today?** Please write down the exact brand name that is written on the container. Please note the full name, including any variant names, fragrance information or the like.  🖉 ……………………….……………………….…………………………………………………….  ……………………….……………………….……………………………………………………. | |
|  | |
| **Do you usually use the same cockpit spray or do you change the brand now and then?**   - I always use the same brand - I switch between different brands. | |
|  | |
| **Is this a cockpit spray from the spray can or a pump spray?** A spray can is a metal can that is under pressure. As long as the spray button is pressed, cockpit spray will come out of the can. For a pump spray you have to build up pressure in the container (often made of glass or plastic) first. This is done by pressing the spray button or a lever / handle. Afterwards only a certain amount will leave the container, no matter how long you press it. | |
| **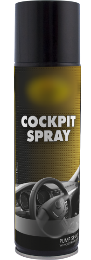**  ⬜ Spray can | 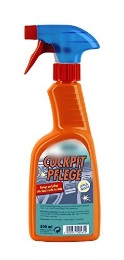  ⬜ Pump spray |
|  | |
| **Please weigh the container of the cockpit spray that you want to use now and enter the displayed weight here.** If possible, use a balance that measures the weight to one gram. Please make sure that the balance shows "0 grams" before the measurement.  Weight before use: 🖉…………………………………. g | |
| **Please take a look at your watch and write down the current time.**  Time at the beginning of the cleaning process: 🖉 ………………………. | |
|  | |
| **Please start to clean the interior of the car now. Once again as a reminder: Please proceed in the same way as you would do without this protocol.** | |

| After using the cockpit spray |
| --- |
|  |
| **If you are done with the application of the cockpit spray for today, please write down the current time.**  Time at the end of the cleaning process: 🖉 ………………………. |
|  |
| **Sometimes one stays in the car for a while after cleaning the interior. Please write down the time when you leave the car. If you left the car right after cleaning the interior, just take the time from the previous question.**  Time when leaving the car: 🖉 ………………………………. |
|  |
| **Please weigh the container of the cockpit spray that you have used and enter the displayed weight here.** Please make sure again that the balance shows "0 grams" before the measurement.  Weight after application: 🖉**………………………………….** g |
|  |
| **What do you estimate: how many puffs did you make today during car care, and how many seconds did any of these puffs last?**  Number of puffs: 🖉…………………….  Average duration of one puff: 🖉…………………… seconds |
| **Where parked your car when you cleaned it today with cockpit spray?**   - Outdoors - In a garage - In a carport - At a different place: 🖉………………………………………………………………… |
|  |
| **Were the car doors open or closed during cleaning?**   - Open ⬜ Closed |
|  |
| **And what about the windows of the car? Were these open or closed during the cleaning process with the cockpit spray?**   - Open ⬜ Closed |
|  |
| **And did you wear gloves when cleaning the interior with cockpit spray or not?**   - Yes, I wore gloves ⬜ No, I did not wear gloves |
|  |
| **And did you wear other protective clothing during the cleaning process with cockpit spray?**   - Yes 🡪 What exactly? 🖉………………………………………………………………………………… - No |
|  |
| **On the container or the packaging of the cockpit spray you can find instructions for use. Did you read them today?**   - Yes, I read them. ⬜ No, I did not read them. |
|  |

| **Did you follow the instructions for use on the container today?**   - Followed instructions🡪 Which instruction did you follow?   🖉 ……………………….……………………….………………………………………  ……………………….……………………….………………………………………  ……………………….……………………….………………………………………  ……………………….……………………….………………………………………   - I did not follow the instructions. | | | | | |
| --- | --- | --- | --- | --- | --- |
|  | | | | | |
| **Please rate the completion of the protocol briefly. Just mark the corresponding number.** | | | | | |
| How interesting was the completion of the protocol on a scale from 1 = "very interesting" to 5 = "not at all interesting" for you? | 1 | 2 | 3 | 4 | 5 |
|  | | | | | |
| How do you rate the length of the protocol on a scale from 1 = "was too long" to 5 = "was too short"? | 1 | 2 | 3 | 4 | 5 |
|  | | | | | |
| How do you rate the comprehensibility of the questions on a scale from 1 = "were understandable" to 5 = "were incomprehensible"? | 1 | 2 | 3 | 4 | 5 |
|  | | | | | |
| How much fun did you have on a scale from 1 = "was fun" to 5 = "was not fun"? | 1 | 2 | 3 | 4 | 5 |
|  | | | | | |
| How elaborate was the participation on a scale of 1 = “not at all complex" to 5 =" very complex"? | 1 | 2 | 3 | 4 | 5 |
|  | | | | | |
| Would you participate in the survey 1 = “again" to 5 = "not participate again"? | 1 | 2 | 3 | 4 | 5 |
| Here is space for further comments / notes to us. | | | | | |

**Thank you for your cooperation!**

Please return the filled-in protocol to us immediately in the attached stamped addressed envelope.
