# Supplementary material for: Consumer behaviour survey for assessing exposure from consumer products: a feasibility study
Source: J Expo Sci Environ Epidemiol. 2018 May 23;29(1):83–94. doi: 10.1038/s41370-018-0040-2 (PMC6760613; doi:10.1038/s41370-018-0040-2)
Supplement: Supplementary file 7 — SI 6 Protocol dishwashing detergent with camera [file 41370_2018_40_MOESM7_ESM.docx]

| Before using the dishwashing detergent – 1. Rinsing process | | |
| --- | --- | --- |
|  | | |
| **Please write down today's date: __ __. __ __. 2016** | | |
|  | | |
| **Which hand dishwashing detergent will you use today?** Please write down the exact brand name that is written on the container. Please note the full name, including any variant names, fragrance information or the like.  🖉 ……………………….……………………….…….……….…….……….……….………………………………… | | |
|  | | |
| **Do you usually use the same hand dishwashing detergent or do you change the brand now and then?**   - I always use the same brand. - I switch between different brands. | | |
|  | | |
| **Please weigh the container of the hand dishwashing detergent that you want to use now and enter the displayed weight here.** If possible, use a balance that measures the weight to one gram. Please make sure that the balance shows "0 grams" before the measurement.  Weight before use. 🖉………………………. g | | |
|  | | |
| To determine the volume of water you use to rinse, we need the dimensions of your sink. If you are using several different sinks, please measure all sinks directly. Sinks are either square, round or oval. Please compare your sink(s) with these sketches and then measure the required values. Please measure only the sink into which the water flows. Please ignore drain basins or areas. | | |
| 1) square | 2) round | 3) oval |
| Length: ………… cm  Width: ………...… cm | Diameter in cm:  …………………….. | Length: ………… cm  Width: ………...… cm |
|  | | |
| **In which sink will you rinse the dishes now?** | | |
| ⬜ sink 1 | ⬜ sink 2 | ⬜ sink 3 |
|  | | |
| **Where did you rinse the dishes today?**   - Kitchen - Other room: 🖉……………………….….….….….….….…………………………. - Please estimate, how big the room is: 🖉……………………….……………sq.m. | | |
|  | | |
| **Will you fill the sink with water or will you do the dishes under running water?**   - I fill the sink with water. 🡪 **Please prepare this now.** - I do the dishes under running water.. - I do something different: 🖉……………………….……………………….……………………….   ……………………….……………………….……………………….………………………………… | | |
|  | | |
| **When you fill the sink with water: After you are finished filling in the water, please place your measuring device vertically in the sink and measure the height of the water.**  Height of the water: ………………………. cm | | |
|  | | |
| **Please take a look at your watch and write down the current time.**  Time at the beginning of the cleaning process: 🖉 ………………………. | | |
|  | | |
| **Please start to wash your dishes now.** | | |

| After using the dishwashing detergent |
| --- |
|  |
| **When you are done with the application of the dishwashing detergent for today, please write down the current time.**  Time at the end of the cleaning process: 🖉 ………………………. |
|  |
| **Please weigh the container of the dishwashing detergent that you used and enter the displayed weight here.** Please make sure again that the balance shows "0 grams" before the measurement.  Weight after application: 🖉………………………. g |

| **Did you wear gloves during the dishwashing process?** | | | |
| --- | --- | --- | --- |
| ⬜ Yes, I wore gloves . | | ⬜ No, I did not wear gloves. | |
|  | | | |
| **Did you also use the hand dishwashing detergent for other purposes in the last week? (e.g. for washing your hands or cleaning surfaces)?** | | | |
| ⬜ Yes | ⬜ No | |  |
|  | | | |
| **On the container or the packaging of the hand dishwashing detergent you can find instructions for use. Did you read them today?** | | | |
| ⬜ Yes, I read them. | ⬜ No, I did not read them. | |  |
|  | | | |
| Did you follow the instructions for use on the container today? (Even if you did not read these instructions this time, it is possible that you know them from previous applications.)   - Followed instructions🡪 Which instruction did you follow?     🖉 ……………………….……………………….………………………………………………………………    ……………………….……………………….………………………………………………………………   - I did not follow the instructions. | | | |

| Before using the dishwashing detergent – 2. Rinsing process | | |
| --- | --- | --- |
|  | | |
| **Please write down today's date: __ __. __ __. 2016** | | |
|  | | |
| **Which hand dishwashing detergent will you use today?** Please write down the exact brand name that is written on the container. Please note the full name, including any variant names, fragrance information or the like.  🖉 ……………………….……………………….…….……….…….……….……….………………………………… | | |
|  | | |
| **Do you usually use the same hand dishwashing detergent or do you change the brand now and then?**   - I always use the same brand. - I switch between different brands. | | |
|  | | |
| **Please weigh the container of the hand dishwashing detergent that you want to use now and enter the displayed weight here.** If possible, use a balance that measures the weight to one gram. Please make sure that the balance shows "0 grams" before the measurement.  Weight before use. 🖉………………………. g | | |
|  | | |
| To determine the volume of water you use to rinse, we need the dimensions of your sink. If you are using several different sinks, please measure all sinks directly. Sinks are either square, round or oval. Please compare your sink(s) with these sketches and then measure the required values. Please measure only the sink into which the water flows. Please ignore drain basins or areas. | | |
| 1) square | 2) round | 3) oval |
| Length: ………… cm  Width: ………...… cm | Diameter in cm:  …………………….. | Length: ………… cm  Width: ………...… cm |
|  | | |
| **In which sink will you rinse the dishes now?** | | |
| ⬜ sink 1 | ⬜ sink 2 | ⬜ sink 3 |
|  | | |
| **Where did you rinse the dishes today?**   - Kitchen - Other room: 🖉……………………….….….….….….….…………………………. - Please estimate, how big the room is: 🖉……………………….……………sq.m. | | |
|  | | |
| **Will you fill the sink with water or will you do the dishes under running water?**   - I fill the sink with water. 🡪 **Please prepare this now.** - I do the dishes under running water.. - I do something different: 🖉……………………….……………………….……………………….   ……………………….……………………….……………………….………………………………… | | |
|  | | |
| **When you fill the sink with water: After you are finished filling in the water, please place your measuring device vertically in the sink and measure the height of the water.**  Height of the water: ………………………. cm | | |
|  | | |
| **Please take a look at your watch and write down the current time.**  Time at the beginning of the cleaning process: 🖉 ………………………. | | |
|  | | |
| **Please start to wash your dishes now.** | | |

| After using the dishwashing detergent |
| --- |
|  |
| **When you are done with the application of the dishwashing detergent for today, please write down the current time.**  Time at the end of the cleaning process: 🖉 ………………………. |
|  |
| **Please weigh the container of the dishwashing detergent that you used and enter the displayed weight here.** Please make sure again that the balance shows "0 grams" before the measurement.  Weight after application: 🖉………………………. g |

| **Did you wear gloves during the dishwashing process?** | | | |
| --- | --- | --- | --- |
| ⬜ Yes, I wore gloves . | | ⬜ No, I did not wear gloves. | |
|  | | | |
| **Did you also use the hand dishwashing detergent for other purposes in the last week? (e.g. for washing your hands or cleaning surfaces)?** | | | |
| ⬜ Yes | ⬜ No | |  |
|  | | | |
| **On the container or the packaging of the hand dishwashing detergent you can find instructions for use. Did you read them today?** | | | |
| ⬜ Yes, I read them. | ⬜ No, I did not read them. | |  |
|  | | | |
| Did you follow the instructions for use on the container today? (Even if you did not read these instructions this time, it is possible that you know them from previous applications.)   - Followed instructions🡪 Which instruction did you follow?     🖉 ……………………….……………………….………………………………………………………………    ……………………….……………………….………………………………………………………………   - I did not follow the instructions | | | |

| Before using the dishwashing detergent – 3. Rinsing process | | |
| --- | --- | --- |
|  | | |
| **Please write down today's date: __ __. __ __. 2016** | | |
|  | | |
| **Which hand dishwashing detergent will you use today?** Please write down the exact brand name that is written the container. Please note the full name, including any variant names, fragrance information or the like.  🖉 ……………………….……………………….…….……….…….……….……….………………………………… | | |
|  | | |
| **Do you usually use the same hand dishwashing detergent or do you change the brand now and then?**   - I always use the same brand. - I switch between different brands. | | |
|  | | |
| **Please weigh the container of the hand dishwashing detergent that you want to use now and enter the displayed weight here.** If possible, use a balance that measures the weight to one gram. Please make sure that the balance shows "0 grams" before the measurement.  Weight before use. 🖉………………………. g | | |
|  | | |
| To determine the volume of water you use to rinse, we need the dimensions of your sink. If you are using several different sinks, please measure all sinks directly. Sinks are either square, round or oval. Please compare your sink(s) with these sketches and then measure the required values. Please measure only the sink into which the water flows. Please ignore drain basins or areas. | | |
| 1) square | 2) round | 3) oval |
| Length: ………… cm  Width: ………...… cm | Diameter in cm:  …………………….. | Length: ………… cm  Width: ………...… cm |
|  | | |
| **In which sink will you rinse the dishes now?** | | |
| ⬜ sink 1 | ⬜ sink 2 | ⬜ sink 3 |
|  | | |
| **Where did you rinse the dishes today?**   - Kitchen - Other room: 🖉……………………….….….….….….….…………………………. - Please estimate, how big the room is: 🖉……………………….……………sp.m. | | |
|  | | |
| **Will you fill the sink with water or will you do the dishes under running water?**   - I fill the sink with water. 🡪 **Please prepare this now.** - I do the dishes under running water.. - I do something different: 🖉……………………….……………………….……………………….   ……………………….……………………….……………………….………………………………… | | |
|  | | |
| **When you fill the sink with water: After you are finished filling in the water, please place your measuring device vertically in the sink and measure the height of the water.**  Height of the water: ………………………. cm | | |
|  | | |
| **Please take a look at your watch and write down the current time.**  Time at the beginning of the cleaning process: 🖉 ………………………. | | |
|  | | |
| **Please start to wash your dishes now.** | | |

| After using the dishwashing detergent |
| --- |
|  |
| **When you are done with the application of the dishwashing detergent for today, please write down the current time.**  Time at the end of the cleaning process: 🖉 ………………………. |
|  |
| **Please weigh the container of the dishwashing detergent that you used and enter the displayed weight here.** Please make sure again that the balance shows "0 grams" before the measurement.  Weight after application: 🖉………………………. g |

| **Did you wear gloves during the dishwashing process?** | | | |
| --- | --- | --- | --- |
| ⬜ Yes, I wore gloves . | | ⬜ No, I did not wear gloves. | |
|  | | | |
| **Did you also use the hand dishwashing detergent for other purposes in the last week? (e.g. for washing your hands or cleaning surfaces)?** | | | |
| ⬜ Yes | ⬜ No | |  |
|  | | | |
| **On the container or the packaging of the hand dishwashing detergent you can find instructions for use. Did you read them today?** | | | |
| ⬜ Yes, I read them. | ⬜ No, I did not read them. | |  |
|  | | | |
| Did you follow the instructions for use on the container today? (Even if you did not read these instructions this time, it is possible that you know them from previous applications.)   - Followed instructions🡪 Which instruction did you follow?     🖉 ……………………….……………………….………………………………………………………………    ……………………….……………………….………………………………………………………………   - I did not follow the instructions | | | |

| **Please rate the completion of the protocol briefly. Just mark the corresponding number.** | | | | | |
| --- | --- | --- | --- | --- | --- |
| How interesting was the completion of the protocol on a scale from 1 = "very interesting" to 5 = "not at all interesting" for you? | 1 | 2 | 3 | 4 | 5 |
|  | | | | | |
| How do you rate the length of the protocol on a scale from 1 = "was too long" to 5 = "was too short"? | 1 | 2 | 3 | 4 | 5 |
|  | | | | | |
| How do you rate the comprehensibility of the questions on a scale from 1 = "were understandable" to 5 = "were incomprehensible"? | 1 | 2 | 3 | 4 | 5 |
|  | | | | | |
| How much fun did you have on a scale from 1 = "was fun" to 5 = "was not fun"? | 1 | 2 | 3 | 4 | 5 |
|  | | | | | |
| How elaborate was the participation on a scale of 1 = “not at all complex" to 5 =" very complex"? | 1 | 2 | 3 | 4 | 5 |
|  | | | | | |
| Would you participate in the survey 1 = “again" to 5 = "not participate again"? | 1 | 2 | 3 | 4 | 5 |
| Here is space for further comments / notes to us. | | | | | |

**Thank you for your cooperation!**

Please return the filled-in protocol to us immediately in the attached stamped addressed envelope.
