# Supplementary material for: Consumer behaviour survey for assessing exposure from consumer products: a feasibility study
Source: J Expo Sci Environ Epidemiol. 2018 May 23;29(1):83–94. doi: 10.1038/s41370-018-0040-2 (PMC6760613; doi:10.1038/s41370-018-0040-2)
Supplement: Supplementary file 8 — SI 7 Protocol filler [file 41370_2018_40_MOESM8_ESM.docx]

| Before using the dishwashing detergent | | |
| --- | --- | --- |
|  | | |
| **Please write down today's date: __ __. __ __. 2017** | | |
|  | | |
| To determine the volume of water you use to rinse, we need the dimensions of your sink. If you are using several different sinks, please measure all sinks directly. Sinks are either square, round or oval. Please compare your sink(s) with these sketches and then measure the required values. Please measure only the sink into which the water flows. Please ignore drain basins or areas. | | |
| 1) square | 2) round | 3) oval |
| Length: ………… cm  Width: ………...… cm | Diameter in cm:  …………………….. | Length: ………… cm  Width: ………...… cm |
|  | | |
| **Where will you rinse the dishes today?**   - Kitchen - Other room: 🖉……………………….….….….….….….…………………………. - Please estimate, how big the room is: 🖉……………………….……………sq.m. | | |
|  | | |
| **Do you usually use the same hand dishwashing detergent or do you change the brand now and then?**   - I always use the same brand. - I switch between different brands. | | |
| Please turn on the camera now and make sure you are recording a video. | | |
|  | | |
| **Before you use the dishwashing detergent, please hold it in the camera for a moment** (Front and back side). | | |
|  | | |
| **Please weigh the container of the hand dishwashing detergent that you want to use now.** **It is important that we can see how heavy the container of detergent was before use.** If possible, use a balance that measures the weight to one gram. Please make sure that the balance shows "0 grams" before the measurement. | | |
|  | | |
| **Please start to wash the dishes now.** During the dishwashing process, please focus the camera at your hands, in order to monitor the exact use of the detergent. We are interested in whether you fill the sink with water or whether you rinse under running water. Of course, combinations are possible as well. | | |
|  | | |
| **If you filled the sink with water:** After you are finished filling in the water, please place your measuring device (ruler, folding rule) vertically in the sink and measure the height of the water.  Again, it is important that you film the measuring device with the camera so that we can read the height of the water. | | |

| After using the dishwashing detergent | | |
| --- | --- | --- |
|  | | |
| **Please weigh the container of the dishwashing detergent that you have used and film the balance while doing so.** Please make sure again that the balance shows "0 grams" before the measurement. | | |
|  | | |
| **Did you also use the hand dishwashing detergent for other purposes in the last week? (e.g. for washing your hands or cleaning surfaces)?** | | |
| ⬜ Yes | ⬜ No |  |
|  | | |
| **On the container or the packaging of the hand dishwashing detergent you can find instructions for use. Did you read them today?** | | |
| ⬜ Yes, I read them. | ⬜ No, I did not read them. |  |
|  | | |
| Did you follow the instructions for use on the container today? (Even if you did not read these instructions this time, it is possible that you know them from previous applications.)   - Followed instructions🡪 Which instruction did you follow?     🖉 ……………………….……………………….………………………………………………………………    ……………………….……………………….………………………………………………………………   - I did not follow the instructions | | |

| **Please rate the completion of the protocol briefly. Just mark the corresponding number.** | | | | | |
| --- | --- | --- | --- | --- | --- |
| How interesting was the completion of the protocol on a scale from 1 = "very interesting" to 5 = "not at all interesting" for you? | 1 | 2 | 3 | 4 | 5 |
|  | | | | | |
| How do you rate the length of the protocol on a scale from 1 = "was too long" to 5 = "was too short"? | 1 | 2 | 3 | 4 | 5 |
|  | | | | | |
| How do you rate the comprehensibility of the questions on a scale from 1 = "were understandable" to 5 = "were incomprehensible"? | 1 | 2 | 3 | 4 | 5 |
|  | | | | | |
| How much fun did you have on a scale from 1 = "was fun" to 5 = "was not fun"? | 1 | 2 | 3 | 4 | 5 |
|  | | | | | |
| How elaborate was the participation on a scale of 1 = “not at all complex" to 5 =" very complex"? | 1 | 2 | 3 | 4 | 5 |
|  | | | | | |
| Would you participate in the survey 1 = “again" to 5 = "not participate again"? | 1 | 2 | 3 | 4 | 5 |
| Here is space for further comments / notes to us. | | | | | |

**Thank you for your cooperation!**

Please return the filled-in protocol and the camera to us immediately in the package that we sent to you. You can use the stamped sticker which we sent to you.
