# Supplementary material for: Consumer behaviour survey for assessing exposure from consumer products: a feasibility study
Source: J Expo Sci Environ Epidemiol. 2018 May 23;29(1):83–94. doi: 10.1038/s41370-018-0040-2 (PMC6760613; doi:10.1038/s41370-018-0040-2)
Supplement: Supplementary file 9 — SI 8 Protocol filler with camera [file 41370_2018_40_MOESM9_ESM.docx]

| Before using the powdery filler |
| --- |
|  |
| **Please write down today's date: __ __. __ __. 2017** |
|  |
| **Which powdery filler will you use today?** Please write down the exact brand name that is written on the container. Please note the full name, including any variant names, fragrance information or the like.  🖉 ……………………….……………………….…………………………………………………….  ……………………….……………………….……………………………………………………. |
|  |
| **Do you usually use the same powdery filler or do you change the brand now and then?**   - I always use the same brand. - I switch between different brands. |
|  |
| **Please weigh the container of the powdery filler that you want to use now and enter the displayed weight here.** If possible, use a balance that measures the weight to one gram. Please make sure that the balance shows "0 grams" before the measurement.  Weight before use. 🖉………………………. g |
|  |
| Mixing of powdery filler with water |
|  |
| **Please start by mixing the filler with water. Once again as a reminder: Please proceed in the same way as you would do without this protocol.** |
|  |
| **Please take a look at your watch and write down the current time.**  Time at the beginning of the mixing process: 🖉 ………………………. |
|  |
| **Please start to mix the filler with water now.** |
|  |
| **If you are done with the mixing of filler with water for today, please write down the current time.**  Time at the end of the mixing process: 🖉 ………………………. |
|  |
| **Please weigh again the container of the powdery filler that you have used and enter the displayed weight here. We want to find out how much filler you have used. If you have used the complete amount of filler in the package, then please write down “0 g”** Please make sure again that the balance shows "0 grams" before the measurement.    Weight after application: 🖉………………………. g |
|  |
| **Did you wear gloves when mixing the filer with water or not?**   - Yes, I wore gloves - No, I did not wear gloves |
|  |
| **If you want to start using the filler now, please take a quick look at your watch and write down the time.**  Time at the beginning of the application: 🖉 ………………………. |
|  |
| **Please start to use the filler now.** |

| After using the filler |
| --- |
| **If you are finished with the entire application of the filler for today, please write down the current time.**  Time at the end of the application 🖉 ………………………. |
|  |
| **Where did you use the filler today?**   - Outdoors - Indoor 🡪 in which room exactly? 🖉……………………….……………………….   🡪 How big is this room? 🖉……………………….……………sq. m. |
|  |
| **How did you apply the filler?**   - With a scraper - With a finishing trowel - With my hands - With something else: 🖉 ……………………….……………………….………….. |
|  |
| **Did you wear gloves when applying filler or not?**   - Yes, I wore gloves ⬜ No, I did not wear gloves |
|  |
| **Did you wear other protective clothing during the application of filler or not?**   - Yes ⬜ No |
| **On the container or the packaging of the filler you can find instructions for use. Did you read them today?**   - Yes, I read them. ⬜ No, I did not read them |
|  |

| **Did you follow the instructions for use on the container today?** (Even if you did not read these instructions this time, it is possible that you know them from previous applications.)   - Followed instructions🡪 Which instruction did you follow?   🖉 ……………………….……………………….………………………………………  ……………………….……………………….………………………………………  ……………………….……………………….………………………………………   - I did not follow the instructions. | | | | | |
| --- | --- | --- | --- | --- | --- |
|  | | | | | |
| **Please rate the completion of the protocol briefly. Just mark the corresponding number.** | | | | | |
| How interesting was the completion of the protocol on a scale from 1 = "very interesting" to 5 = "not at all interesting" for you? | 1 | 2 | 3 | 4 | 5 |
|  | | | | | |
| How do you rate the length of the protocol on a scale from 1 = "was too long" to 5 = "was too short"? | 1 | 2 | 3 | 4 | 5 |
|  | | | | | |
| How do you rate the comprehensibility of the questions on a scale from 1 = "were understandable" to 5 = "were incomprehensible"? | 1 | 2 | 3 | 4 | 5 |
|  | | | | | |
| How much fun did you have on a scale from 1 = "was fun" to 5 = "was not fun"? | 1 | 2 | 3 | 4 | 5 |
|  | | | | | |
| How elaborate was the participation on a scale of 1 = “not at all complex" to 5 =" very complex"? | 1 | 2 | 3 | 4 | 5 |
|  | | | | | |
| Would you participate in the survey 1 = “again" to 5 = "not participate again"? | 1 | 2 | 3 | 4 | 5 |
| Here is space for further comments / notes to us. | | | | | |

**Thank you for your cooperation!**

Please return the filled-in protocol to us immediately in the attached stamped addressed envelope.
