# Supplementary material for: Consumer behaviour survey for assessing exposure from consumer products: a feasibility study
Source: J Expo Sci Environ Epidemiol. 2018 May 23;29(1):83–94. doi: 10.1038/s41370-018-0040-2 (PMC6760613; doi:10.1038/s41370-018-0040-2)
Supplement: Supplementary file 10 — SI 9 Protocol paints and lacquers [file 41370_2018_40_MOESM10_ESM.docx]

| Before using the powdery filler |
| --- |
|  |
| **Please write down today's date: __ __. __ __. 2017** |
|  |
| **Which powdery filler will you use today?** Please write down the exact brand name that is written on the container. Please note the full name, including any variant names, fragrance information or the like.  🖉 ……………………….……………………….…………………………………………………….  ……………………….……………………….……………………………………………………. |
|  |
| **Do you usually use the same powdery filler or do you change the brand now and then?**   - I always use the same brand. - I switch between different brands. |
|  |
| **Please weigh the container of the powdery filler that you want to use now and enter the displayed weight here.** If possible, use a balance that measures the weight to one gram. Please make sure that the balance shows "0 grams" before the measurement.  Weight before use. 🖉………………………. g |
|  |

| Mixing of powdery filler with water |
| --- |
|  |
| **Please put on the camera now, switch it on and make sure that the camera is running.** |
|  |
| **First, we need pictures of the powdery filler you will be using. Please turn the container in front of the camera if possible at a distance where you can read the most important information.** |
|  |
| **Please start to mix the filler with water now. Once again as a reminder: Please proceed in the same way as you would do without this protocol.** |
|  |
| **Please weigh again the container of the powdery filler that you have used and enter the displayed weight here. We want to find out how much filler you have used. If you have used the complete amount of filler in the package, then please write down “0 g”** Please make sure again that the balance shows "0 grams" before the measurement.  Weight after mixing with water: 🖉………………………. g |
|  |
| **Did you wear gloves when mixing the filer with water or not?**   - Yes, I wore gloves - No, I did not wear gloves |
|  |
| **Did you wear other protective clothing during the mixing of filler or not?**   - Yes 🡪 What exactly? 🖉………………………………………………………………………………… - No |
|  |
| **Please start to use the filler now.** |

| After using the filler |
| --- |
|  |
| **Where did you use the filler today?**   - Outdoors - Indoor 🡪 in which room exactly? 🖉……………………….……………………….   🡪 How big is this room? 🖉……………………….……………sq. m. |
|  |
| **How did you apply the filler?**   - With a scraper - With a finishing trowel - With my hands - With something else: 🖉 ……………………….……………………….………….. |
|  |
| **Did you wear gloves when applying filler or not?**   - Yes, I wore gloves - No, I did not wear gloves |
|  |
| **Did you wear other protective clothing during the application of filler or not?**   - Yes - No |
|  |
| **On the container or the packaging of the filler you can find instructions for use. Did you read them today?**   - Yes, I read them - No, I did not read them |

| **Did you follow the instructions for use on the container today?** (Even if you did not read these instructions this time, it is possible that you know them from previous applications.)   - Followed instructions🡪 Which instruction did you follow?   🖉 ……………………….……………………….………………………………………  ……………………….……………………….………………………………………  ……………………….……………………….………………………………………   - I did not follow the instructions. | | | | | |
| --- | --- | --- | --- | --- | --- |
|  | | | | | |
| **Please rate the completion of the protocol briefly. Just mark the corresponding number.** | | | | | |
| How interesting was the completion of the protocol on a scale from 1 = "very interesting" to 5 = "not at all interesting" for you? | 1 | 2 | 3 | 4 | 5 |
|  | | | | | |
| How do you rate the length of the protocol on a scale from 1 = "was too long" to 5 = "was too short"? | 1 | 2 | 3 | 4 | 5 |
|  | | | | | |
| How do you rate the comprehensibility of the questions on a scale from 1 = "were understandable" to 5 = "were incomprehensible"? | 1 | 2 | 3 | 4 | 5 |
|  | | | | | |
| How much fun did you have on a scale from 1 = "was fun" to 5 = "was not fun"? | 1 | 2 | 3 | 4 | 5 |
|  | | | | | |
| How elaborate was the participation on a scale of 1 = “not at all complex" to 5 =" very complex"? | 1 | 2 | 3 | 4 | 5 |
|  | | | | | |
| Would you participate in the survey 1 = “again" to 5 = "not participate again"? | 1 | 2 | 3 | 4 | 5 |
| Here is space for further comments / notes to us. | | | | | |

**Thank you for your cooperation!**

Please return the filled-in protocol and the camera to us immediately in the package that we have sent to you. You can use the stamped sticker which we have sent to you.
