# Supplementary material for: Consumer behaviour survey for assessing exposure from consumer products: a feasibility study
Source: J Expo Sci Environ Epidemiol. 2018 May 23;29(1):83–94. doi: 10.1038/s41370-018-0040-2 (PMC6760613; doi:10.1038/s41370-018-0040-2)
Supplement: Supplementary file 11 — SI 10 Protocol paints and lacquers with camera [file 41370_2018_40_MOESM11_ESM.docx]

| Before using paints/lacquers |
| --- |
|  |
| **Please write down today's date: __ __. __ __. 2017** |
|  |
| **Which paint or lacquer will you use today?** Please write down the exact brand name that is written on the container. Please note the full name, including any variant names, fragrance information or the like.  🖉 ……………………….……………………….…………………………………………………….  ……………………….……………………….……………………………………………………. |
|  |
| **Do you usually use the same paint or lacquer or do you change the brand now and then?**   - I always use the same brand. - I switch between different brands |
|  |
| **Which object do you want to paint today? if you want to paint a picture, or if you want to do something else with it, we are also interested of course.**   - Painted object: 🖉 …………….………………………….…………………………...… - I have painted a picture. - I did something else: 🖉 …………….………………………….………………………….………………………….…………… |
|  |
| **Please weigh the container of the paint or lacquer that you want to use now and enter the displayed weight here. If you want to use several paints / lacquers, please repeat the weighing for all containers.** If possible, use a balance that measures the weight to one gram. Please make sure that the balance shows "0 grams" before the measurement.  Weight before use (container 1). 🖉………………………. g  Weight before use (container 2). 🖉………………………. g  Weight before use (container 3). 🖉………………………. g  Weight before use (container 4). 🖉………………………. g |
|  |
| **Please take a look at your watch and write down the current time.**  Time at the beginning of the painting process: 🖉 ………………………. |
|  |
| **Please start using the paint or lacquer now. Once again as a reminder: Please proceed in the same way as you would do without this protocol.** |

| After using paints/lacquers | | | | | |
| --- | --- | --- | --- | --- | --- |
| **If you are finished with the entire application of the paint/lacquer for today, please write down the current time.**  Time at the end of the application 🖉 ………………………. | | | | | |
|  | | | | | |
| **Please weigh the containers of the paints or lacquers that you have used today and enter the displayed weight here.** Please make sure again that the balance shows "0 grams" before the measurement.  Weight after use (container 1). 🖉………………………. g  Weight after use (container 2). 🖉………………………. g  Weight after use (container 3). 🖉………………………. g  Weight after use (container 4). 🖉………………………. g | | | | | |
|  | | | | | |
| **Where did you use the paint/lacquer today?**   - Outdoors - Indoor 🡪 in which room exactly? 🖉……………………….……………………….   🡪 How big is this room? 🖉……………………….……………sq. m. | | | | | |
|  | | | | | |
| **In case, the application took place indoor: How long have you been in the same room after you finished painting?**  🖉 ………………………. minutes | | | | | |
|  | | | | | |
| **In case, the application took place indoor: Were the doors open or closed during application of the paint?**   - Doors open ⬜ Doors closed | | | | | |
|  | | | | | |
| **In case, the application took place indoor: Were the windows open or closed during application of the paint?**   - Windows open ⬜ Windows closed | | | | | |
|  | | | | | |
| **Did you wear gloves while painting or not?**   - Yes, I wore gloves ⬜ No, I did not wear gloves | | | | | |
|  | | | | | |
| **Did you wear other protective clothing during painting or not?**   - Yes 🡪 What exactly? 🖉 ……………………….……………………….………………………. - No | | | | | |
|  | | | | | |
| **On the container or the packaging of the paint or lacquer you can find instructions for use. Did you read them today?**   - Yes, I read them. ⬜ No, I did not read them. | | | | | |
| **Did you follow the instructions for use on the container today?** (Even if you did not read these instructions this time, it is possible that you know them from previous applications.)   - Followed instructions🡪 Which instruction did you follow?   🖉 ……………………….……………………….………………………………………  ……………………….……………………….………………………………………  ……………………….……………………….………………………………………   - I did not follow the instructions. | | | | | |
|  | | | | | |
| **Please rate the completion of the protocol briefly. Just mark the corresponding number.** | | | | | |
| How interesting was the completion of the protocol on a scale from 1 = "very interesting" to 5 = "not at all interesting" for you? | 1 | 2 | 3 | 4 | 5 |
|  | | | | | |
| How do you rate the length of the protocol on a scale from 1 = "was too long" to 5 = "was too short"? | 1 | 2 | 3 | 4 | 5 |
|  | | | | | |
| How do you rate the comprehensibility of the questions on a scale from 1 = "were understandable" to 5 = "were incomprehensible"? | 1 | 2 | 3 | 4 | 5 |
|  | | | | | |
| How much fun did you have on a scale from 1 = "was fun" to 5 = "was not fun"? | 1 | 2 | 3 | 4 | 5 |
|  | | | | | |
| How elaborate was the participation on a scale of 1 = “not at all complex" to 5 =" very complex"? | 1 | 2 | 3 | 4 | 5 |
|  | | | | | |
| Would you participate in the survey 1 = “again" to 5 = "not participate again"? | 1 | 2 | 3 | 4 | 5 |
| Here is space for further comments / notes to us. | | | | | |

**Thank you for your cooperation!**

Please return the filled-in protocol to us immediately in the attached stamped addressed envelope.
